# The equilibrium between antagonistic signaling pathways determines the number of synapses in *Drosophila*

**DOI:** 10.1371/journal.pone.0184238

**Published:** 2017-09-11

**Authors:** Sheila Jordán-Álvarez, Elena Santana, Sergio Casas-Tintó, Ángel Acebes, Alberto Ferrús

**Affiliations:** Institute Cajal C.S.I.C., Madrid, Spain; Institut de genomique fonctionnelle, FRANCE

## Abstract

The number of synapses is a major determinant of behavior and many neural diseases exhibit deviations in that number. However, how signaling pathways control this number is still poorly understood. Using the *Drosophila* larval neuromuscular junction, we show here a PI3K-dependent pathway for synaptogenesis which is functionally connected with other previously known elements including the Wit receptor, its ligand Gbb, and the MAPkinases cascade. Based on epistasis assays, we determined the functional hierarchy within the pathway. Wit seems to trigger signaling through PI3K, and Ras85D also contributes to the initiation of synaptogenesis. However, contrary to other signaling pathways, PI3K does not require Ras85D binding in the context of synaptogenesis. In addition to the MAPK cascade, Bsk/JNK undergoes regulation by Puc and Ras85D which results in a narrow range of activity of this kinase to determine normalcy of synapse number. The transcriptional readout of the synaptogenesis pathway involves the Fos/Jun complex and the repressor Cic. In addition, we identified an antagonistic pathway that uses the transcription factors Mad and Medea and the microRNA bantam to down-regulate key elements of the pro-synaptogenesis pathway. Like its counterpart, the anti-synaptogenesis signaling uses small GTPases and MAPKs including Ras64B, Ras-like-a, p38a and Licorne. Bantam downregulates the pro-synaptogenesis factors PI3K, Hiw, Ras85D and Bsk, but not AKT. AKT, however, can suppress Mad which, in conjunction with the reported suppression of Mad by Hiw, closes the mutual regulation between both pathways. Thus, the number of synapses seems to result from the balanced output from these two pathways.

## Introduction

Synapses are dynamic neural structures that can exhibit a turnover of hours [1–3] and are influenced by day/light cycle, age, and learning [4–8] among other physiological processes. Molecular mechanisms that could account for building/removal of synapses within these short times are largely unknown. Changes in synapse number, even if relatively small, can elicit notable changes in behavior [9, 10], or result in neural disorders [11]. For example, a mild 16% loss of inhibitory synapses has been reported in the cortex of schizophrenia patients [12]. Also, subtle perceptual and cognitive impairments manifest as a consequence of changes in the number of synapses well before cells are definitively lost by irreversible neurodegeneration [13, 14].

Extensive previous studies have identified proteins and processes that mediate synapse development and turnover. These include neurotrophins and neuropeptides [15–17], trafficking of receptors to and from the synapse [18], components such as N-cadherin [19], CaMKII [20, 21] or PSD-95 [22]. Also, synapse activity contributes to refine the number of synapses in a relatively long term basis [23, 24]. However, prior to these mechanisms of synapse maintenance, a different set of signals are required to initiate synaptogenesis and to cancel it when the appropriate number of them is reached. Different neuron types have characteristic numbers of synapses. In general, neuron size and synapse number correlate positively [25, 26]. Nonetheless, the neuron-type specific number of synapses, “the correct number”, can fluctuate according to normal physiology, and these fluctuations can occur in the range of hours [27, 28]. Several proteins have been reported to signal for synaptogenesis in *Drosophila* [2, 29, 30] and vertebrates [31–33]. However, their hierarchy has not been established yet, nor a regulatory mechanism that could account for a rapid change in the number of synapses has been proposed.

As it is well known, different signaling pathways may share components depending on the cell type, physiological process or developmental context [34, 35]. For example, PI3K has been identified in multiple pathways downstream of various receptor types including tyrosine-kinase, G-protein-coupled and integrins [36, 37]. This scenario justifies the need to study a signaling pathway in a defined cell type rather than assuming that interactions described in a given context will apply to all cell types. Also, the biological relevance of molecular interactions requires to be validated under *in vivo* conditions. Here, we set out to identify the up- and down-stream components from PI3K, a key factor that we previously identified as a modulator of synaptogenesis in identified *Drosophila* neurons [2] and which is conserved in mammalian hippocampal cells [32]. We revisited other elements known to be involved in synaptogenesis, and analyzed their functional hierarchy by epistasis assays. In the course of our study, however, we identified and characterized an antagonistic signaling that counterbalances the pro-synaptogenesis pathway. Both pathways cross-regulate each other suggesting that the final number of synapses result from the equilibrium between their respective signaling activities.

Neurites, varicosities (a.k.a. boutons) and synapses are morphologically different features in a neuron and, consequently, the signaling for their genesis is also different. For example, while AKT promotes collateral branching and synapse formation [2, 38], FAK reduces branching but promotes synapses [39]. Also, small GTPases show differential localization in postsynaptic spines *vs* axon collateral branches [40]. Even collaterals from the same axon exhibit different ways of branching (reviewed in [41, 42]). These facts, justify to count synapses directly in this study, rather than using bouton number as a proxy.

## Material and methods

### Fly stocks

The class I phosphoinositide kinase PI3K is represented in *Drosophila* by Dp110. All fly constructs used here correspond to Dp110 but, for simplicity, we refer to them as PI3K. The pan-neural driver *elav-Gal4* (w*; P{w[+mC] = GAL4-elav.*L}3*) [43] and those affecting motor neurons, D42-Gal4 (w*; P{w[+mW.*hs] = GawB}D42)* [44], as well as lines *myc-PI3K (w*^*1118*^*;P{w[+mC] = Myc-Dp110}1)*, *myc-PI3K*^*ΔRBD*^ (*w*^*1118*^*;P{w[+mC] = Myc-Dp110[RBD]}1*), *UAS-PI3K*^*92E*.*CAAX*^ *(P{w[+mC] = Dp110-CAAX}1*, *y*^*1*^ w*) [45], UAS-Bsk (w*; P{w[+mC] = UAS-bsk.*B}2)*, *UAS-Bsk*^*DN*.^ (*w*^*1118*^ *P{w[+mC] = UAS-bsk*.*DN}2*) [46], *UAS-hep* (*w*^[^*^]^*; P{w[+mC] = UAS-hep*.*B}2*) [47], *UAS-htl* (*y*^*1*^ w*; P{w[+mC] = UAS-htl.*M}YYDFR-F16*), *htl*^*AB42*^ (w*; htl^*AB42*^*red e/TM3*, *P{ry[+t7*.*2] = ftz/LacZ}SC1*, *ry*^*RK*^ *Sb*^*1*^ *Ser*^*1*^), *btl*^*dev1*^ (*btl*^*dev1*^*/TM1*, *T(2;3)D*^*7*^, *red*^*1*^ *D*^*7*^) [48], *UAS-Fos* (*w*^*1118*^*; P{w[+mC] = UAS-Fra}2*), *UAS-Jun* (*y*^*1*^ *w*^*1118*^*; P{UAS-Jra}2*) [49], *UAS-Fos*^*DN*^ (*w*^*1118*^*; P{w[+mC] = UAS-Fra*.*Fbz}5*), *UAS-Jun*^*DN*^ (*w*^*1118*^*; P{w[+mC] = Jbz}1*) [50], *UAS-Ras85D* (w*; P{w[+mC] = UAS-Ras85D.*K}5–1*), *UAS-Ras*^*DN*^ (*P{w[+mC] = UAS-ras*.*N17}TL1*, *w*^*1118*^) [51, 52], UAS-Ras64B (w[*]; P{w[+mC] = UAS-Ras64B.*V14}1)*, *UAS-Ask1*^*RNAi*^ *(y*^*1*^ sc* v^*1*^*; P{y[+t7*.*7] v[+t1*.*8] = TRiP*.*GL00238}attP2)*, *UAS-Mad*^*RNAi*^ (*y*^*1*^ *v*^*1*^*; P{y[+t7*.*7] v[+t1*.*8] = TRiP*.*JF01263}attP2*), *UAS-Licorne (y*^*1*^ *w*^*67c23*^ *P{y[+t7*.*7] = Mae-UAS*.*6*.*11}lic[GG01785])*, *UAS-Rala*^*DN*^ (w*; P{w[+mC] = UAS-Rala.*S25N}2*), *UAS-Slpr*^*RNAi*^ (*y*^*1*^ sc* *v1**;* *P{TRiP*.*HMS00742}attP2*), *UAS-put*^*RNAi*^ *(**y*^*1*^ sc* *v1**;* *P{TRiP*.*HMS01944}attP40*), *actin-Gal4* (*P{Act5C-GAL4}*) and *tub*^*LL7*^*-Gal4* (*P{tubP-GAL4}*) were from Bloomington Stock Center (NIH P40OD018537) (http://flystocks.bio.indiana.edu/). The other driver used in motor neurons, *OK6-Gal4*, was a gift from Cahir J. O’Kane (University of Cambridge, United Kingdom) [53]. Strains *UAS-PI3K (y*^*1*^ *w*^*1118*^*;P{w[+mC] = UAS-PI3K92E*.*Exel}2*) and *UAS-PI3K*^*DN*^ (*y*^*1*^ w*; P{w[+mC] = Dp110[D954A]}2) [54] were provided by Dr. J. Botas (Baylor College of Medicine, Houston, TX). Strains *UAS-Hiw* (*P{UAS-hiw*.*W}*) [55], *UAS-wit* (*P{UAS-wit*.*M}*), *wit*^*B11*^ (*bw*^*1*^*; wit*^*B11*^ *st*^*1*^*/TM6B*, *Tb*^*1*^), *wit*^*A12*^ (*bw*^*1*^*; wit*^*A12*^ *st*^*1*^*/TM6B*, *Tb*^*1*^) [56], *UAS-gbb* (*P{UAS-gbb*.*K}*) [57], *gbb*^*1*^, *gbb*^*2*^ [58] *UAS-Mad*^*#2A3*^ and *UAS-Medea*^*#5*.*13A3*^ [59] were a gift from Dr. G. Marqués (University of Minnesota). Strains *UAS-Wnd* (w*; P{w[+mC] = UAS-wnd.*C}2*) (50), H*iw*^*ND8*^ (w* hiw^*ND8*^) [60] and H*iw*^*ΔE3*^ (55) were kindly provided by Dr. A. DiAntonio (University of Washington in St. Louis, USA). Driver *Mhc-Gal4* (*P{Mhc-GAL4*.*U}*) [61] was used to target the postsynaptic cell. The *puc*^*RNAi*^ (GD3018), *p38a*^*RNAi*^ (KK102484), *Medea*^*RNAi*^ (GD19689) and *Mad*^*RNAi*^ (KK110517) strains were from the Vienna Stock Center (http://stockcenter.vdrc.at/control/main). The microRNA *UAS-bantamA* strain (*M{UAS-ban*.*S}ZH-86Fb*) [62] was provided by Dr. M. Milán (IRB, Barcelona, Spain), and line *UAS-cic(3xHA) att86Fb* (F001848) was from Dr. G. Jiménez (IBMB, Barcelona).

### Validation of genetic tools and procedures

For down-expression experiments, mutants or dominant negative forms were used when available. In a few cases, RNAi constructs were employed as an alternative. All RNAi lines used in this study do not have reported off-targets. In addition, their effectiveness was tested with general Gal4 drivers (*gmr-Gal4*, *tub*^*LL7*^*-Gal4* or *actin-Gal4*) under the criterion of morphological or adult viability phenotypes. Furthermore, some genetic combinations were also tested for protein effects in Western blots (see main Text). The RNAi that, having passed the previous criteria, failed to yield a synaptic phenotype constitute an innocuous RNAi control (see S1 Table). The synaptic value of reference corresponds to genotypes in which the Gal4 drives neutral *UAS-GFP* or *UAS-LacZ* constructs. To count synapses, the *D42-Gal4* driver was used throughout, while for qPCR assays, we used the *elav-Gal4*. All synapse counts correspond to the larval motor neuron 6/7 from abdominal segment A3. Only one neuromuscular junction per crawling LIII larvae was used in order to maximize the stochastic variability and, hence, to increase the biological relevance of the statistical differences. Cultures were kept at 25°C and reared under non-crowding conditions, 10–15 males and 20–30 females per vial changed every other day. All genotypes were obtained from crosses repeated over a two years period.

### Experimental cell system

The neuromuscular junction (NMJ) of third larval instar [63] was used as experimental system. Boutons are often used as a surrogate of synapses in quantitative studies of synaptogenesis. However, common experience shows that synapses are not confined to boutons, and boutons can be devoid of synapses. To avoid these caveats, we adopted the criterion of counting the total number of mature active zones per neuromuscular junction irrespective of their bouton allocation. Genetic manipulations were driven to selected motor neurons using the binary Gal4/UAS system [64]. Synapses were visualized under confocal microscopy by the monoclonal antibody nc82 (1:10, DSHB, IA) which identifies the Bruchpilot protein, homolog to the mammalian CAST [65], and serves as an active zone-specific marker [2, 66–68] located at the edge of the characteristic T bar specialization of fly synapses [69]. The neuronal membrane was labeled with rabbit anti-HRP (1:200, Jackson ImmunoResearch). The secondary antibodies used were Alexa 488 (goat anti-mouse, 1:500, Molecular Probes) and Alexa 568 (goat anti-rabbit, 1:500, Molecular Probes). Larvae were mounted in Vectashield (Vector Labs).

### Image acquisition and synapse number quantification

Confocal Images were acquired with a Leica Confocal Microscope TCS SP5 II (Mannheim, Germany). Synapse number was determined in all genotypes using the IMARIS software (Bitplane). Since IMARIS performs 3D reconstructions of the neuromuscular junction, the identification of synapses can be considered as reliable. Finally, to avoid misidentification of synapses, a threshold was stablished and a quality filter of shape and size was applied in all cases to unambiguously count synapses independently of changes of branching, length or area in the selected motor neurons. To stablish the threshold, we choose a confocal plane to measure the size of the nc82-positive spots present in that plane irrespective of whether they are contained within a bouton or along the axon. These measurements provide an average value that, for the acquisition conditions at 63x magnification (1024x256 pixels), corresponds to 0.5μm. This value is consistent with the synapse size as determined by transmission electron microscopy, and was fed to the software to count rounded spots of that size. Parameters were kept constant throughout the study of all genotypes.

### Quantitative PCR

mRNA extractions from third instar larvae were obtained through standard protocols using TRIZOL (Invitrogen) and kept frozen at -80°C. SuperSCript II Reverse Transcriptase (*Invitrogen*) was used with 5 μg of mRNA of each sample following the manufacturer instructions. Quantification of gene expression was determined using specific Taqman probes (*Applied Biosystems*). Each reaction was done in triplicate and with at least three different samples of each genotype.

### Western blotting

For biochemical assays, 5–10 fly heads were homogenized in 20 μl of lysis buffer containing NaCl 150 mM, 0.05% Tween-20 and TBS pH 7.5. For detection of pAKT protein, approximately 5–10 head equivalents of protein extract were analyzed on a 4%–12% gradient SDS-PAGE and electro-blotted onto nitrocellulose 0.45 μM (GE Healthcare) 100V for 1 h. The membranes were blocked in 5% BSA in TBS-Tween-20 buffer and incubated with pAKT antibody (1:3000) (Cell Signaling) and anti-tubulin (1:10000) (Sigma-Aldrich) overnight at 4°C with constant agitation. The antibody-antigen interaction was visualized by chemiluminescence using HRP-coupled secondary antibody and developed with ECL (Pierce).

### Statistics

Statistical significance was calculated using one way ANOVA test. Synapse number comparisons were always performed between experimental conditions *vs* their corresponding control in each experiment. Female larvae were used throughout unless otherwise indicated. Data are plotted as whisker diagrams indicating the maximum and minimum values (vertical line), the 25% and 75% values (box) and the median value (horizontal line within the box). An ANOVA test was used also to compare mRNA levels in qPCR assays. The software GraphPad Instat 3 was used throughout. Significant differences between groups were indicated by *p<0.05, **p<0.001 and ***p<0.0001.

## Results

Synapses were visualized from the presynaptic active zone (AZ) as nc82 immuno-positive spots which identifies the CAST-type protein Brp. These spots are located opposite to glutamate receptors (GluR) [3, 70] (S1A Fig). The levels of Brp have been related to the type of neurotransmitter release mechanism, spontaneous *vs* inducible [71]. In our study we do not make a distinction between these two types of AZs. A 3D reconstruction of a normal A3 NMJ is shown in S1 Movie. Using the binary UAS/Gal4 expression system [64], we manipulated genetically the motor neuron that innervates muscles 6/7 of the larval abdominal segment A3. For brevity, the symbols ↓ and ↑ are used to indicate elicited down- and over-expression, respectively. Genotypes are indicated in the figure legends and synapse number raw data are shown in S2 Table.

### Receptors and ligands in the pro-synaptogenesis pathway

The receptor Wit and its ligand Gbb modulate the number of boutons and their growth [72–74]. Boutons and synapses, however, are two different neuronal processes which are not causally related (see below). Thus, we assayed here the potential relationship of PI3K with Gbb and Wit in the context of synaptogenesis.

**Fig 1:**
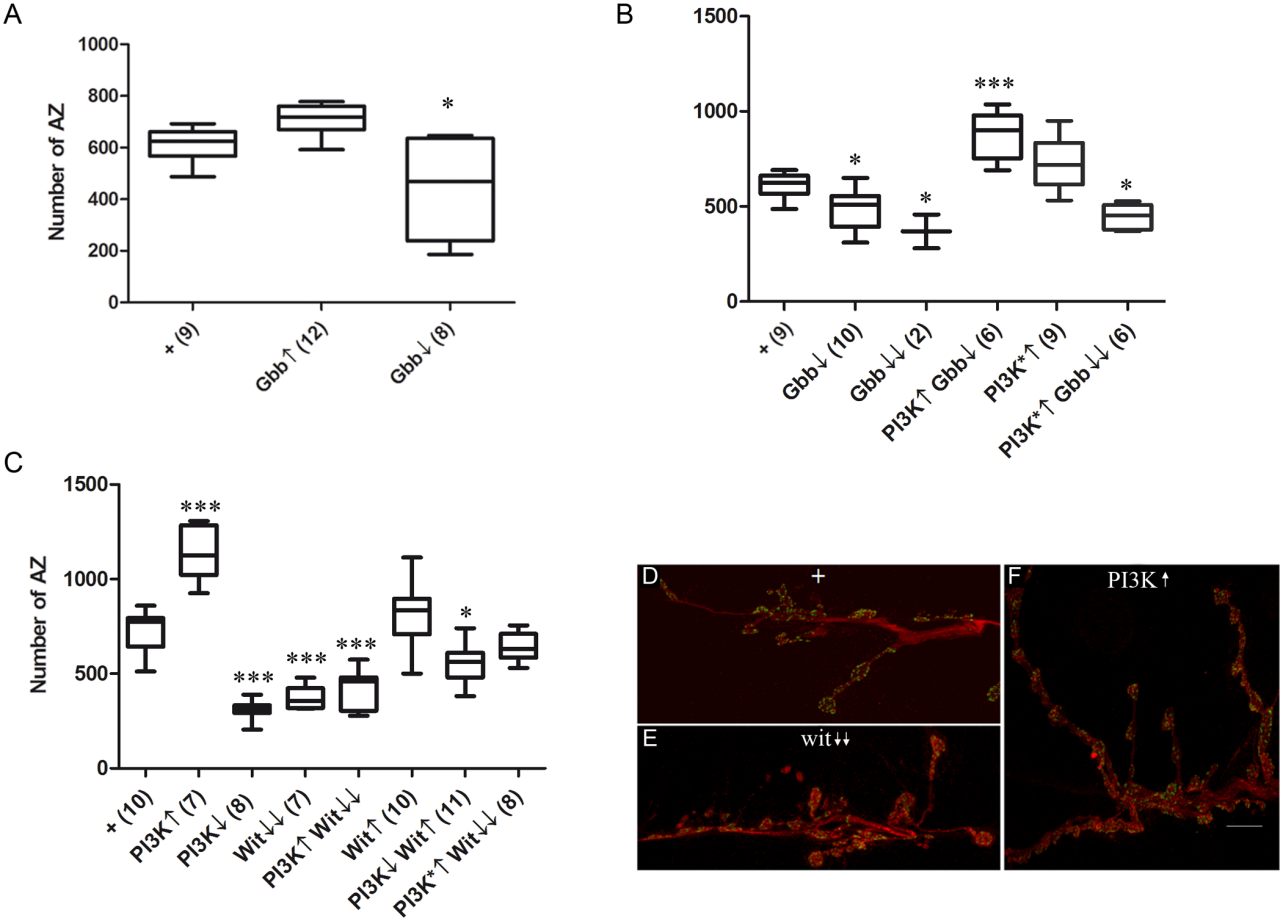
Ligand and receptor of the pro-synaptogenesis pathway. **A)** The over- (↑) or down-expression (↓) of the ligand Gbb in the muscle elicits mild changes in the number of synapses as indicated by the number of active zones (AZ) viewed as nc82 spots. Genotypes: **+** = *Mhc-Gal4/+*. **Gbb↑** = *Mhc-Gal4/+;UAS-gbb/+*. **Gbb↓** = *Mhc-Gal4/+;UAS-gbb*^*RNAi*^*/+*. **B)** The *gbb* mutant larvae, either in heterozygous (Gbb↓) or homozygous (Gbb↓↓) conditions show reduced number of synapses. These data support a role of this ligand in synaptogenesis. Note, however, that synapses have not been eliminated (see main text). The viability of homozygotes is very poor and only two LIII larvae could be obtained. This is evidence that Gbb plays additional developmental roles beyond synaptogenesis. The up-regulation of the native form of PI3K suppresses the synapse effect in the heterozygotes although the effect of PI3K on its own is somewhat reduced (compare with PI3K**↑** in panel C). The constitutively active PI3K, PI3K*, improves the viability of the homozygous *gbb* null mutants but the mutant synapse number is only weakly recovered, and it remains clearly below normal (see main text). Genotypes: + = *D42-Gal4/+*. **Gbb↓** = *gbb*^*1*^/+*; D42-Gal4/+*. **Gbb↓↓** = *gbb*^*1*^*/gbb*^*2*^*; D42-Gal4/+*. **PI3K↑Gbb↓** = *UAS-PI3K*/*gbb*^*1*^; *D42-Gal4/+*. PI3K*↑ = UAS-PI3K*/+; D42-Gal4/+. PI3K*↑ Gbb↓↓ = UAS-PI3K*/+; gbb^*1*^*/gbb*^*2*^*; D42-Gal4/+*. **C)** The up-regulation of the native form of PI3K (PI3K↑) increases the number of synapses while its down-regulation (PI3K**↓**) yields the opposite effect. The overexpression of the receptor Wit is ineffective for synapse formation while the mutant condition (Wit**↓↓)** strongly reduces synapses. Note, however, that neither Wit nor Gbb depletion can eliminate synapses fully. Combinations of Wit and PI3K show that the mutant condition for Wit prevails over the overexpression of PI3K suggesting that PI3K requires activation by Wit. Consistent with this notion, the downregulation of PI3K still maintains its phenotype of reduced synapse number even though Wit is over-expressed. In the same line, a constitutively active form of PI3K, PI3K*, suppresses the mutant condition for the receptor. These results support the conclusion that PI3K is functionally downstream from the receptor Wit. Genotypes: **+** *= D42-Gal4/+* pooled with *OK6-Gal4/+*. **PI3K↑** = *UAS-PI3K/+*; *D42-Gal4/+*. **PI3K***↓* = *UAS-PI3K*^*DN*^*/+; D42-Gal4/+*. **Wit↓↓** = *OK6-Gal4/+; wit*^*A12*^*/wit*^*B11*^. **PI3K↑Wit↓↓** = *OK6-Gal4/UAS-PI3K*; *wit*^*A12*^*/wit*^*B11*^. **Wit↑** = *D42-Gal4/UAS-Wit*. **PI3K↓Wit↑** = *UAS-PI3K*^*DN*^*/+; D42-Gal4/UAS-Wit*. PI3K*↑Wit↓↓ = UAS-PI3K*/+; OK6-Gal4/+; wit^*A12*^*/wit*^*B11*^. **D-F)** Representative images of motor neuron 6–7 from larval abdominal segment A3 in normal (+) (**D**), *wit*^*A12*^*/wit*^*B11*^ (↓) (**E**) and *D42-Gal4>UAS-PI3K* (↑) (**F**) genotypes. Number of NMJ analyzed in independent larvae is indicated in parenthesis adjacent to the genotype. Bar in **F** = 10μm.

The ligand Gbb is expressed in, and secreted from, the muscle [59, 75]. The selective over-expression of Gbb in the muscle (*Mhc-Gal4*) had no effect on the number of AZ in the genetically normal presynaptic motor neuron (Gbb↑, Fig 1A). This lack of effect may reflect a down-titration of the excess of Gbb by Crimpy [73, 76] although we did not explore this possibility further. The *gbb* attenuation by an RNAi (Gbb↓), however, reduces AZ number (Fig 1A). Heterozygous mutant larvae for *gbb* exhibit a mild reduction of synapses (Fig 1B). The homozygous mutants are lethal before LIII stage, preventing synapse counting. In the very few *gbb*^*1*^*/gbb*^*2*^ larvae that could be obtained, the number of AZ was 30% reduced (Gbb↓↓) (Fig 1B). The relatively mild reduction of synapses, even in the homozygotes, suggests that Gbb is not the only ligand, or Wit is not the only receptor, for synaptogenesis (see below and Discussion). The up-regulation of PI3K in the motor neuron suppresses these *gbb* phenotypes, including the poor viability of the hetero-allelic combination (Fig 1B). This suppression places PI3K functionally down-stream from Gbb. We also tested a constitutively active form of PI3K, PI3K*, which rescued the viability trait of the homozygous *gbb* mutants (Fig 1B). The synapse reduction trait, however, was not fully rescued, perhaps due to the relatively minor pro-synaptogenesis effect of the PI3K* construct itself. This difference between PI3K and PI3K* constructs could result from their relative levels of expression due to their chromosomal sites of insertion, and/or stability of the proteins, among other reasons. Nevertheless, PI3K* proved more effective in the rescue of the mutant genotype of the canonical Gbb receptor, Wit (see below and Discussion).

The receptor Wit is a key player for neuromuscular junction (NMJ) development [53, 56]. Eliminating its expression (hetero-allelic combination *wit*^*A12*^*/wit*^*B11*^) results in decrease of AZ numbers (Wit↓↓, Fig 1C). This effect supports the role of Wit as a key receptor in synaptogenesis. The co-expression of PI3K↑ yields the phenotype of Wit↓↓ alone, but a constitutive active form of PI3K, PI3K*, can suppress the Wit↓↓ phenotype (Fig 1C). On the other hand, the over-expression of Wit is ineffective to change AZ numbers and the co-downexpression with PI3K results in the phenotype of PI3K↓ alone (Fig 1C). All these data indicate that PI3K is functionally downstream from Wit and requires its activation by the receptor.

The functional link of PI3K with Wit, considered as a receptor Ser/Thr kinase type (RSTK), appears unusual, since PI3K is more often related to receptors of the Tyr kinase type (RTK) (see Discussion). To further look into this issue, we tested classical RTKs in the context of synaptogenesis (S1 Table). No changes in AZ numbers were observed, underlying the specificity of Wit in neurons. In particular, the lack of effect of Thick veins (Tkv) and Saxophone (Sax), either separately or in combination, indicates that, contrary to NMJ development or growth [72], the role of Wit to signal for synaptogenesis does not rely on Tkv or Sax heterodimers. Among the tested RTKs, we included Torso which is not normally expressed in motor neurons. This ectopic expression was also ineffective to elicit synaptogenesis which further underlies the specificity of PI3K activation by the receptor Wit in the context of synaptogenesis.

Signaling from virtually all RTK pathways debouch in the relive of target gene silencing imposed by the transcriptional repressor Capicua (CIC) which is degraded [77]. We reasoned that, if Wit signals through a RTK pathway, then an increase of CIC levels should mimic the effects of Wit↓. Driving *UAS-cic* to motor neurons (*Gal4-D42*) caused a severe reduction of the number of synapses almost identical to that of Wit↓ (S1B Fig). These results open the possibility that Wit could elicit several modes of signaling depending on binding ligand or oligomerization partner (see Discussion).

Representative images of NMJs showing normal (+), increased (↑) and decreased (↓) AZ numbers illustrate how bouton number, or bouton size, do not correlate with synapse number **(**Fig 1D–1F, see also S1 Movie to illustrate AZs outside boutons and boutons without AZs). This is one, among several others (see below), argument that justify the need to count synapses directly rather than using bouton number as a surrogate to evaluate synapse number changes (see Materials and methods).

Two other RTKs were also investigated due to their postsynaptic (muscle) localization, Heartless (Htl) and Breathless (Btl) [78–80]. Mutant heterozygotes for each of these two FGFR-like receptors show a significant increase in the number of synapses (S1C Fig), indicating that they play a role in synaptogenesis. Thus, we tested the possible involvement of Htl and Btl on *gbb* expression by qPCR assays. The reduction of Htl (heterozygous *htl*^*AB42*^*/+*) increased *gbb* transcription while that of Btl (heterozygous *btl*^*dev1*^*/+*) led to the opposite effect (S2A Fig), suggesting that the two receptors regulate Gbb levels through antagonistic signaling within the muscle. To test if there could be a reverse, neuron-to-muscle, signaling to regulate *gbb*, we carried out additional qPCR assays in larvae with neuronal (*elav-Gal4*) up- or down-expression of PI3K. No transcriptional changes in *gbb* were detected in these larvae (S2A Fig). Thus, the reported activity of Gbb in neurons is not functionally related to PI3K levels. Our data are consistent with the proposal of two distinct pools of Gbb to control synapse structure and function, respectively [76].

In addition, we assayed the transcriptional expression of the known ligands of Htl and Btl; Pyramus (Pyr), Thisbe (Ths) and Branchless (Bnl), respectively [81–84] (S2B–S2D Fig). No effect on the expression of these ligands was observed when PI3K was altered in neurons; which is consistent with the muscular origin of Gbb signaling for synaptogenesis. So far, the data show that, once Gbb is secreted from the muscle and synaptogenesis is triggered in the motor neuron, no feed-back signaling is elicited. That is, once initiated, synaptogenesis proceeds autonomously in the motor neuron. Synapse activity, however, may require Gbb mediated coordination from the postsynaptic side as proposed [76]. Retrograde signaling has been reported in activity dependent modulation of synapses where it is mediated by BMP [85]. Here, we focus on synapse formation rather than on activity.

### Pro-synaptogenesis signaling downstream from the receptor

**Fig 2:**
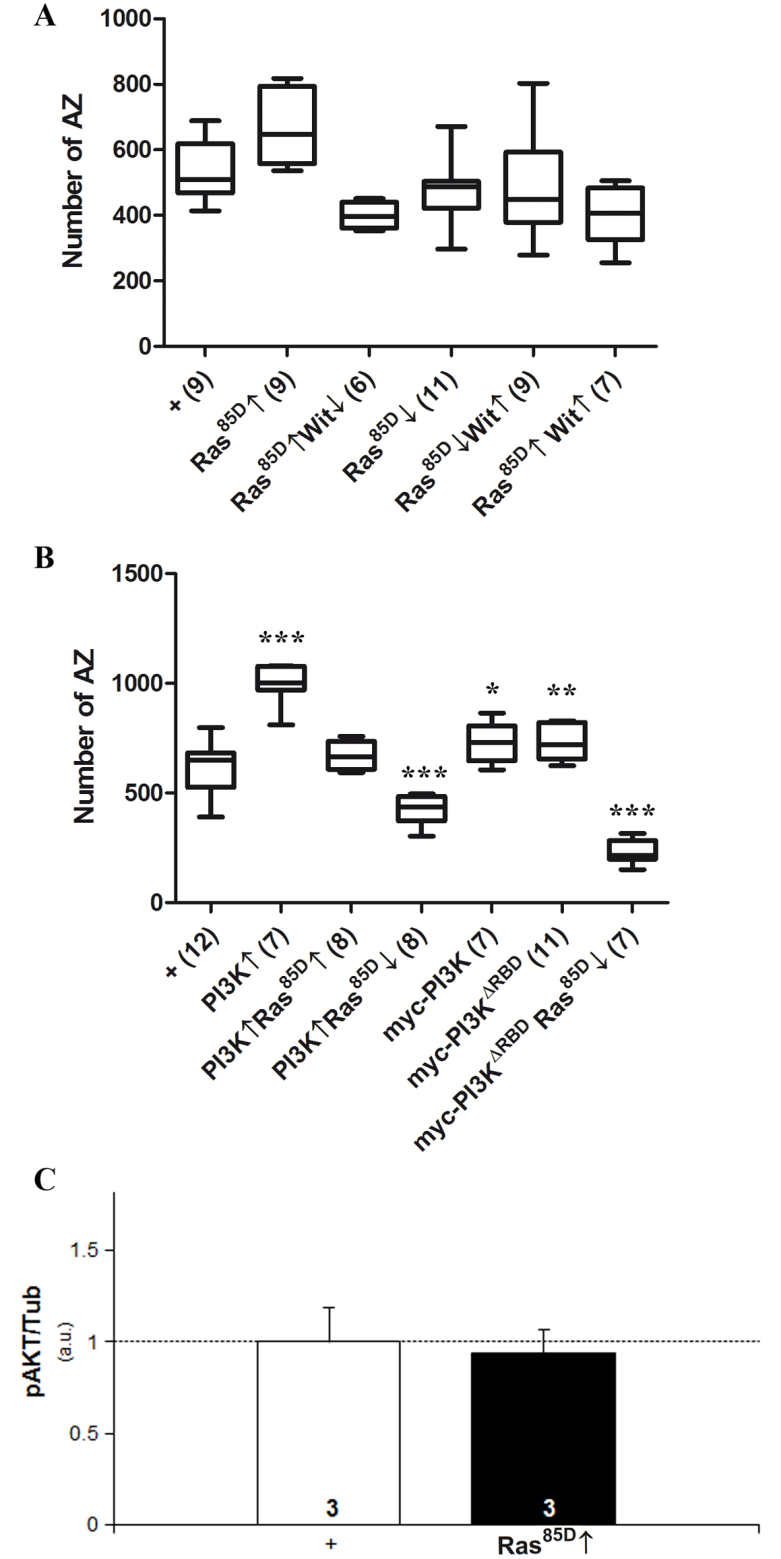
Signaling factors downstream from the receptor Wit. **A)** The over- or down-expression of Ras85D, either alone or in combination with manipulations of Wit, seem ineffective to change synapse number. Further experiments, however, proved the contrary (see below). Genotypes: **+** = *D42-Gal4/+*. **Ras85D↑** = *UAS-Ras85D/+*; *D42-Gal4/+*. **Ras85D↑Wit↓** = *UAS-Ras85D/+*; *D42-Gal4/UAS-Wit*^*RNAi*^. **Ras85D↓** = *UAS-Ras85D*^*DN*^*/+; +/+; D42-Gal4/+*. **Ras85D↓Wit↑** = *UAS-Ras85D*^*DN*^*/+; UAS-Wit /+; D42-Gal4/+*. **Ras85D↑Wit↑** = *UAS-Wit/UAS-Ras85D; D42-Gal4/+*. **B**) PI3K-Ras85D interactions. Both, the over- or down-expression of Ras85D, which seemed ineffective on the synapse number, suppress the synapse increase due to PI3K↑ when jointly expressed. In the case of Ras85D↓ the phenotype is converted into synapse decrease. These features demonstrate that Ras85D is functionally related to PI3K in the context of synaptogenesis. The up-regulation of a PI3K form that is deleted in the Ras phosphorylation site, *myc-PI3K*^ΔRBD^, is still able of increase synapses even though its control, *myc-PI3K*, is somewhat less effective than PI3K. The *PI3K*^ΔRBD^ form, as the normal PI3K, transforms the Ras85D↓ phenotype into a severe synapse reduction. These data are consistent with Ras85D being functionally located somewhere down stream in the PI3K pathway. Genotypes: **+** = *D42-Gal4/+*. **PI3K↑** = *UAS-PI3K/+; D42-Gal4/+*. **PI3K↑Ras85D↑ =** *UAS-Ras85D/+; UAS-PI3K/+; D42-Gal4/+*. **PI3K↑Ras85D↓** = *UAS-Ras85D*^*DN*^*/+; UAS-PI3K/+; D42-Gal4/+*. **myc-PI3K↑** = *D42-Gal4/UAS-myc-Dp110*. **myc-PI3K**^**ΔRBD**^**↑** = *D42-Gal4/UAS-myc-Dp110*^*RBD*^. **myc-PI3K**^**ΔRBD**^**↑Ras85D↓** = *UAS-Ras85D*^*DN*^*/+; D42-Gal4/UAS-myc-Dp110*^*RBD*^. **C)** Western blot analysis (triplicates) of phospho-AKT relative levels under Ras85D overexpression. No increase of pAKT is detected demonstrating that Ras85D is functionally downstream from AKT. Genotypes: **+** = *D42-Gal4/+*. **Ras85D↑** = *UAS-Ras85D/+*; *D42-Gal4/+*.

Since, in addition to tyrosine-kinase, G-protein coupled receptors can activate PI3K, and the small GTPase Ras has been implicated in vertebrate synaptogenesis [86], we tested its *Drosophila* homolog, Ras85D, in the Wit/PI3K signaling context. In the first series of experiments, neither the overexpression of Ras85D nor its downregulation by a dominant negative form, Ras85D^DN^, revealed any effect on synapse number (Fig 2A). Also, co-manipulation of Ras85D and Wit in either direction or combination failed to show synapse number effects. Although these data could be taken as evidence that Ras85D does not play a role in *Drosophila* synaptogenesis, in contrast to vertebrates, the subsequent series of experiments with PI3K proved the opposite.

The simultaneous up-regulation of Ras85D and PI3K suppressed the synapse number increase due to PI3K alone (Fig 2B). This result demonstrates that the *UAS-Ras85D* construct is effective and that both proteins are functionally linked in the context of synaptogenesis. Furthermore, the *UAS-Ras85D*^*DN*^ construct (Ras85D↓) is also effective because it transforms the synapse increase due to PI3K↑ into a significant decrease (Fig 2B). Thus, consistent with vertebrates, *Drosophila* Ras85D is also involved in synaptogenesis, and we can add that it acts by counteracting the role of PI3K. Since, in certain pathways, PI3K needs Ras binding for maximal activity [45], we considered PI3K as a potential site of convergence with Ras85D in the context of synaptogenesis. To determine if the Ras85D requirement occurs at the PI3K step, we tested a form of PI3K in which the Ras binding site is deleted, PI3K^ΔRBD^. This myc-tagged PI3K^ΔRBD^ form elicited a synapse increase equivalent to that of the normal PI3K (myc-PI3K) demonstrating that Ras binding is dispensable for PI3K activity in this context (Fig 2B). Consistent with the functional link between Ras85D and PI3K, the down regulation of Ras85D did suppress the pro-synaptic effect of PI3K^ΔRBD^ yielding a strong decrease (Fig 2B).

As a further attempt to locate Ras85D in the signaling hierarchy, we tested by Western blot the relative levels of phospho-AKT in flies over-expressing Ras85D [87]. The data show that the mechanism of Ras85D activity does not involve an increase of p-AKT levels (Fig 2C). Therefore, we can conclude that, contrary to other signaling contexts, PI3K-Ras85D binding is not a requirement in synaptogenesis, placing the role of Ras85D in synaptogenesis further down along the pathway.

### Intermediate signaling in the pro-synaptogenesis pathway

**Fig 3:**
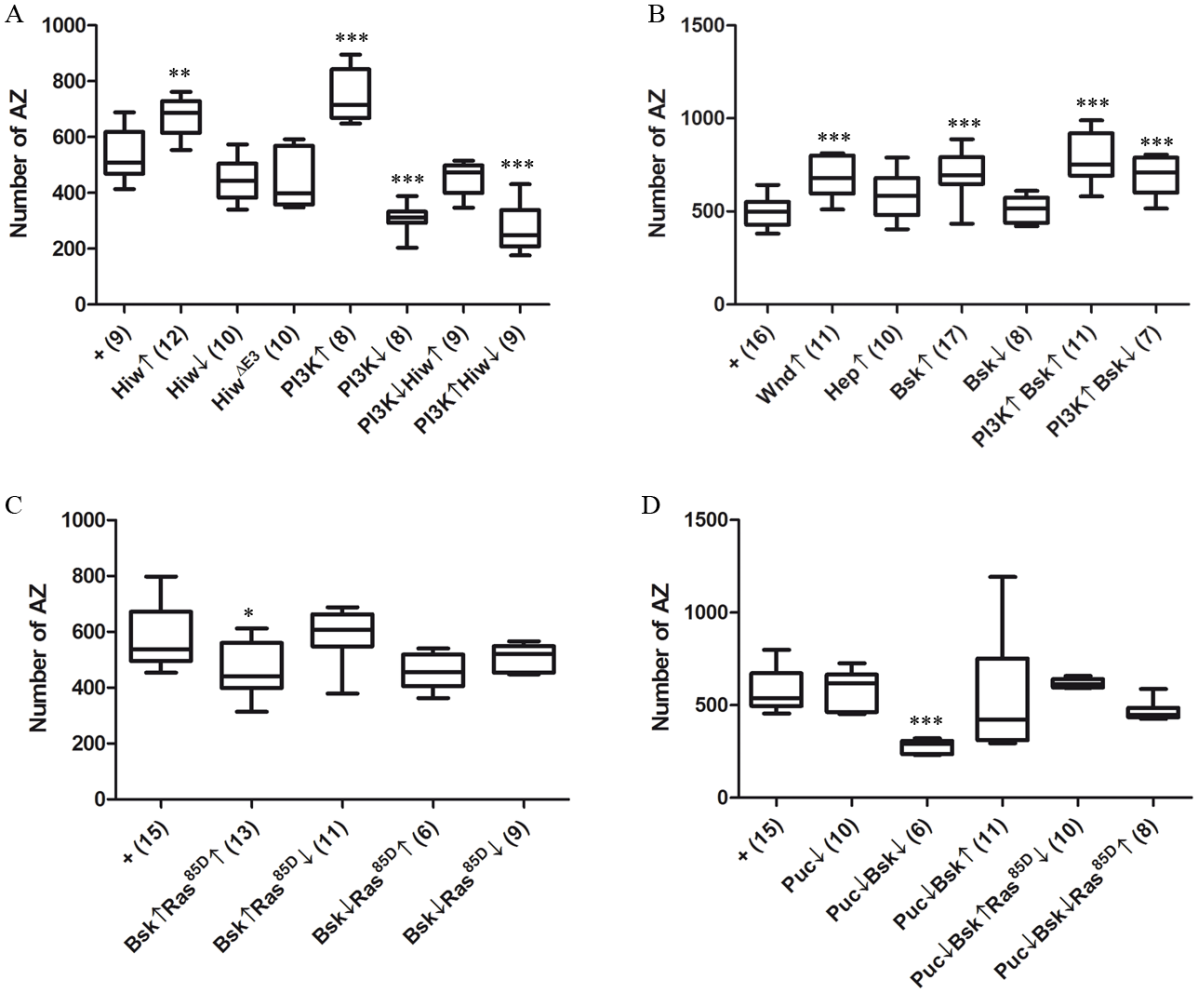
Intermediate signaling in the pro-synaptogenesis pathway. **A**) Interactions between Hiw and PI3K. The overexpression (Hiw↑) increases the number of synapses, while the mutant *hiw*^*ND8*^ or a form deleted in the E3 domain, Hiw^ΔE3^, show a non-significant tendency towards reduction only. However, while the over-expression of PI3K (PI3K↑) increases the number of synapses and its downregulation with a dominant negative form (PI3K↓) reduces it, both effects are suppressed by manipulating Hiw in the opposite directions. These data place Hiw functionally downstream from PI3K in the pathway. Genotypes: **+** = *D42-Gal4/+*. **Hiw↑** = *UAS-Hiw/+; D42-Gal4/+*. **Hiw***↓* = males *hiw*^*ND8*^*; +/+; D42-Gal4/+*. **Hiw**^**ΔE3**^**↑** = *D42-Gal4/UAS-NT-Hiw*^*ΔRING*^. **PI3K↑** = *UAS-PI3K/+; D42-Gal4/+*. **PI3K***↓* = *UAS-PI3K*^*DN*^*/+; D42-Gal4/+*. **PI3K***↓***Hiw↑** = *UAS-Hiw/UAS-PI3K*^*DN*^*; D42-Gal4/+*. **PI3K↑Hiw***↓* = males *hiw*^*ND8*^*; UAS-PI3K/+; D42-Gal4/+*. **B**) The MAPKs of the pathway. Wnd shows the expected pro-synaptogenesis effect while Hep fails to reach statistical significance. However, their target, Bsk/JNK, does show the expected synapse increase. Apparently, the Bsk↓ construct is ineffective and there is no evidence of synergy or antagonism with PI3K in the PI3K↑Bsk↑ or PI3K↑ Bsk↓ conditions (however, see below). Genotypes: **+** = *D42-Gal4/+*. **Wnd↑** = *UAS-Wnd/+; D42-Gal4/+*. **Hep↑** = *UAS-Hep/+; D42-Gal4/+*. **Bsk↑** = *UAS-Bsk/+; D42-Gal4/+*. **Bsk↓** = *D42-Gal4/UAS-Bsk*^*DN*^. **PI3K↑Bsk↑** = *UAS-Bsk/UAS-PI3K; D42-Gal4/+*. **PI3K↑Bsk↓** = *UAS-PI3K/+; D42-Gal4/UAS-Bsk*^*DN*^. **C)** The possible regulation of Bsk by Ras85D. Although the up-regulation of Bsk had proven pro-synaptogenic, its concomitant over-expression with the seemingly ineffective Ras85D transforms the pro- into an anti-synaptogenesis effect. Also, the apparently ineffective Ras85D↓ suppresses the pro-synaptogenic effect of Bsk↑. These data are indicative of a regulatory interaction between Ras85D and Bsk. The Bsk↓ condition, which proved ineffective, remained unchanged irrespective of the two possible alterations of Ras85D. Genotypes: **+** = *D42-Gal4/+*. **Bsk↑Ras**^**85D**^**↑** = *UAS-Bsk/UAS-Ras85D; D42-Gal4/+*. **Bsk↑Ras**^**85D**^**↓** = *UAS-Ras85D*^*DN*^*/+; UAS-Bsk/+; D42-Gal4/+*. **Bsk↓Ras**^**85D**^**↑** = *UAS-Ras85D/+; D42-Gal4/UAS-Bsk*^*DN*^. **Bsk↓Ras**^**85D**^**↓** = *UAS-Ras85D*^*DN*^*/+; +/+; D42-Gal4/UAS-Bsk*^*DN*^. **D)** The Bsk regulator Puc and the convergence with Ras85D in a tripartite interaction. Whereas the downregulation of Puc or Bsk separately yields no effect on synaptogenesis, their joint co-downregulation results in a strong decrease of the number of synapses. Also, the synapse increase elicited by Bsk↑ is rendered non-significant because of the wide dispersion of values elicited by the joint down regulation of Puc. The two triple combinations tested result in a notable reduction of the variability in the number of synapses (see text). Together, the data in **C** and **D** reveal a complex regulatory network at the final step of the MAPK cascade. Genotypes: **+** = *D42-Gal4/+*. **Puc↓** = *UAS-puc*^*RNAi*^/+; *D42-Gal4/+*. **Puc↓Bsk↓** = *UAS-puc*^*RNAi*^/*+; D42-Gal4/UAS-Bsk*^*DN*^. **Puc↓Bsk↑** = *UAS-Bsk/UAS-puc*^*RNAi*^*; D42-Gal4/+*. **Puc↓Bsk↑Ras**^**85D**^**↓** = *UAS-Ras85D*^*DN*^*/+; UAS-puc*^*RNAi*^/*UAS-Bsk; D42-Gal4/+*. **Puc↓Bsk↓Ras**^**85D**^**↑** = *UAS-Ras85D*, *UAS-puc*^*RNAi*^*/+; D42-Gal4/ UAS-Bsk*^*DN*^.

Highwire (Hiw) has been reported as a negative regulator of NMJ growth in *Drosophila* [50, 59] and *C*. *elegans* [88]. This interpretation is based on the observation that *Hiw* mutants in both species show overgrown NMJs. Hiw has, at least, two different substrates: Wallenda, the first MAPK of the JNK cascade [50], and the transcriptional factor Medea [44, 89]. We tested the involvement of PI3K in the Hiw-MAPK signaling (Fig 3A).

As expected, the Hiw↑ condition increased significantly the number of synapses. The loss-of-function allele *Hiw*^*ND8*^ (Hiw↓) or the overexpression of a form deleted in the E3 ubiquitin ligase domain, *Hiw*^*Δ3*^, yielded only a trend towards synapse number reduction that failed to reach statistical significance (Fig 3A). However, as in the case of Ras85D above, the phenotypic effects of the loss-of-function condition for Hiw became evident when tested in combinations with PI3K. The co-expression of Hiw↑ and PI3K↓ results in the mutual suppression of the single phenotypes and yields normal number of synapses (Fig 3A). The opposite combination, Hiw↓PI3K↑, transforms the PI3K↑ into a significant reduction of synapse number; thus, revealing the expected effect of Hiw↓ (Fig 3A). A further evidence of the Hiw-PI3K interaction is reflected in the synergy of the Hiw↓PI3K↓ combination which results in lethality earlier than the third larval instar. This feature prevented synapse counting. Taken together, these results are consistent with the functional location of Hiw downstream from PI3K. Interestingly, the *Hiw*^*ND8*^ mutant exhibited the reported increase in branch length [55, 60], which contrasts with the lack of effect on synapse number (S3 Table). This is another example illustrating the independence of neuritogenesis with respect to synaptogenesis. The case of Hiw is also in line with a previous report on LIMK1 which binds the Wit receptor to promote synapse stability, but the binding is dispensable for synapse growth or function [90].

The *Drosophila* MAPKs *Wallenda (Wnd)*, *Hemipterous (Hep)* and *Basket (bsk)* are homologues of vertebrate DLK (MAPK), MKK7 (MAPKK) and JNK (MAPKKK), respectively. Their role in synaptogenesis and their relationship with Hiw are relatively well established [50], albeit complex. The context-dependence of the Hiw-MAPKs signaling is illustrated by the recently reported cell autonomous and non-cell autonomous effects of Hiw. Whereas Hiw attenuates Wnd, Bsk and the transcription factor Jun (see below) in the neuron, in glial cells, the transcriptional output of Hiw seems to be through Fos, which acts on adjacent neurons via an unidentified secreted factor [91]. Here, we confirmed most of the expected MAPK effects in the NMJ with increases in synapse number after Wnd and Bsk upregulations (Fig 3B).

In the case of Hep, its upregulation does not seem to produce the expected synapse increase (Fig 3B). The homologue in vertebrates, MKK7, cooperates with MKK4 to activate JNK/Bsk [92]. This cooperative effect is non-redundant and it exhibits a wide repertoire of context-dependent signaling properties [93]. Thus, it is plausible that the fly Hep would require a MKK4 equivalent to display its synaptogenesis effects. On the issue of neuritogenesis *versus* synaptogenesis (see above), it is worth noting that Wnd↑ and Hep↑ yielded the reported branch overgrowth (S3A–S3C Fig) [49, 50], but synapses were increased in Wnd↑ only. Likewise, Hiw↓ increased branching (S3D Fig**),** but not synapses. This suggests that neurito- and synaptogenesis share some signaling steps while they keep others as specific.

Concerning Bsk, the ↑ condition yielded the expected synapse increase but the ↓ condition seemed ineffective (Fig 3B). However, as in previous cases, Bsk↓ revealed its effect on synaptogenesis when combined with other regulatory elements of the pathway. The joint up-regulation of PI3K and Bsk, two factors promoting synapse increase, maintain their single phenotypes without evidences of synergy (Fig 3B). This is consistent with the location of both factors in the same pathway. Further, if Bsk would be down stream of PI3K in a simple functional dependency, one would expect that Bsk↓ would suppress PI3K↑. This double genotype still showed synapse increase, although not as high as PI3K↑ alone (Fig 3B). This result suggests that the actual output from Bsk is subject to the regulation by additional factors beyond PI3K and the tested MAPKs.

Since we had concluded (see above) that Ras85D is functionally located downstream from PI3K, we considered if Ras85D could contribute to the pathway through a regulatory convergence on Bsk. Thus, we tested the joint ↓ and ↑ conditions for Bsk and Ras85D. The synapse number increase caused by Bsk↑ is transformed into reduction by Ras85D↑ which, by itself, had no effect (Fig 3C). This transformation of phenotype is suggestive of antagonistic regulation between both enzymatic activities or upon a third substrate. Consistent with this interpretation, the Bsk↑ phenotype is cancelled by Ras85D↓ yielding normal synapse number (Fig 3C). The other two combinations possible, whose single components had no synaptic effect, remained ineffective (Fig 3C).

Beyond the novel regulatory role of Ras85D over Bsk proposed here, Bsk has been shown to be regulated also by the phosphatase *puckered* (*puc*) in epithelial cells [94]. We tested if the synaptogenic effects of Bsk could be modified by Puc. Although Puc↓ and Bsk↓ had no effect on synapses separately, the double combinations resulted in strong reduction of synapse number, indicating that Puc is also a Bsk regulator in this context (Fig 3D). As reasoned above, uncovering a phenotype by the combination of two elements which appeared ineffective on their own is an indication that both elements may be part of a binary system of regulation. Furthermore, the seemingly ineffective Puc↓ condition suppresses the effect of Bsk↑ by dispersing the values of synapse number beyond statistical significance (Fig 3D).

To evaluate if the regulatory roles of Ras85D and Puc over Bsk are independent or redundant, we assayed triple combinations of these constructs. Puc↓ does not modify the suppression that Ras85D↓ plays over Bsk↑ already (Fig 3C and 3D). Also, Puc↓ does not modify the lack of effects exhibited by Bsk↓ and Ras85D↑, either separately or in combination. Taken together, these data may indicate that Bsk and Ras85D/Puc antagonize each other in the context of synaptogenesis, perhaps by helping to set the normal activity threshold of Bsk. In this way, Bsk activity level is revealed as a particularly sensitive step in the signaling pathway and subject to the dual, but independent, regulation by Puc and Ras85D.

### Effectors of the pro-synaptogenesis pathway

AP-1 is a transcriptional complex that includes the heterodimer Jun/Fos [95]. It is known to play multiple roles in development and, in conjunction with the MAPK cascade, it is involved in neuronal growth [49, 96] and hence, in neural plasticity and memory [97, 98]. Actually, *puc* seems to modulate target gene expression by antagonizing a Rho GTPase pathway that stabilizes AP-1, perhaps through a kinase-dependent mechanism [99].

**Fig 4:**
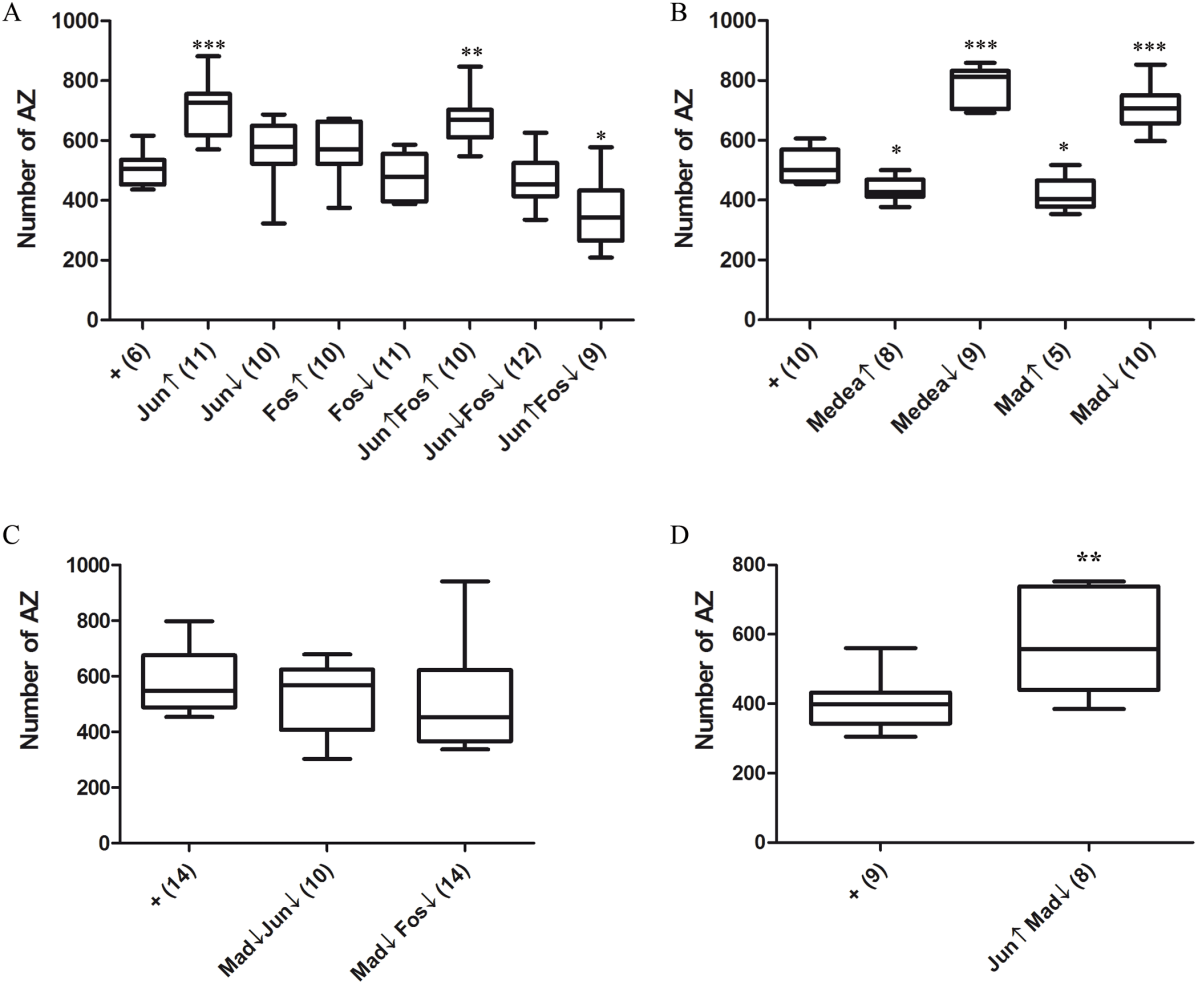
Effectors of the pathway. **A)** Epistasis analysis of the AP-1 components, Fos and Jun. Single manipulations of these two transcription factors in either direction yields the expected synapse increase in the case of Jun↑ only, which could be interpreted as if the other three constructs were ineffective. However, Fos↓ transforms the Jun↑ synapse increase effect into a decrease. This and other results throughout this study demonstrate that the seemingly ineffective constructs are functional. Likely, Jun and Fos play their role through interactions with other anti-synaptogenesis transcription factors (see below). Genotypes: **+** = *D42-Gal4/+*. **Jun↑** = *D42-Gal4/UAS-Jun*. **Jun↓** = *UAS-Jun*^*DN*^*/+; D42-Gal4/+*. **Fos↑** = *UAS-Fos/+; D42-Gal4/+*. **Fos↓** = *UAS-Fos*^*DN*^*/+; D42-Gal4/+*. **Jun↑Fos↑** = *UAS-Fos/+; D42-Gal4/ UAS-Jun*. **Jun↓Fos↓** = *UAS-Fos*^*DN*^*/UAS-Jun*^*DN*^*; D42-Gal4/+*. **Jun↑Fos↓** = *UAS-Fos*^*DN*^*/+; D42-Gal4/UAS-Jun*. **B)** The Med-Mad components. The up- or down-regulation of these two transcription factors yield effects consistent with a role as negative regulators of synaptogenesis which implies the existence of an anti-synaptogenesis pathway. Genotypes: **+** = *D42-Gal4/+*. **Medea↑** = *D42-Gal4/UAS-Medea*^*#5*.*13A3*^. **Medea↓** = *D42-Gal4/UAS-TRiP*.*JF02218attP*. **Mad↑** = *UAS-Mad*^*#2A2*^*/D42-Gal4/+*. **Mad↓** = *UAS-Mad*^*RNAi*^ */+; D42-Gal4/+*. **C)** Mad interactions with AP-1. The apparently ineffective Jun↓ and Fos↓ constructs suppress the Mad↓ phenotype when jointly expressed. This set of data proves that the constructs are effective and that Fos and Jun interact with the anti-synaptogenesis factor Mad. Genotypes: **+** = *D42-Gal4/+*. **Mad↓Jun↓ =** *UAS-Mad*^*RNAi*^ */UAS-Jun*^*DN*^*; D42-Gal4/+*. **Mad↓Fos↓ =** *UAS-Fos*^*DN*^*/UAS-Mad*^*RNAi*^*; D42-Gal4/+*. **D)** In a separate experiment, hence the reason for a separate panel, two conditions that had shown pro-synaptogenesis effects, Jun↑ and Mad↓, although they still show synapse increase, the magnitude of the effect is lower than each element separately. This is suggestive of an antagonistic interaction between these two transcription factors. Genotypes: **+** = *D42-Gal4/+*. **Jun↑Mad↓ =** *UAS-Mad*^*RNAi*^ */+; D42-Gal4/UAS-Jun*.

We tested the relative contributions of Jun and Fos to synaptogenesis. Out of the four genotypes possible, only Jun↑ gave a phenotype, synapse increase, which justifies its qualification as a pro-synaptogenesis element (Fig 4A). As in the case of Ras85D above, the seemingly ineffective constructs revealed their role when in combination with other elements. The joint overexpression of Jun and Fos maintained the synapse increase due to Jun↑ alone. However, the effect of the later becomes transformed into synapse reduction by the co-expression with the apparently ineffective Fos↓ (Fig 4A).

As reasoned for the case of Bsk and Ras85D above, this result with Jun↑Fos↓ suggests a functional antagonism between Jun and Fos in the context of synaptogenesis, as if the two components of the AP-1 complex would counterbalance each other. The role of Fos as transcriptional activator has been shown to be context dependent, in particular the context provided by Jun and Mad [100] (see also below on the synaptic effects of Mad). If the regulation of AP-1 stability by Puc and a Rho GTPase turns out to be effective in the context of synaptogenesis as proposed [99], it is likely that revealing synapse phenotypes would require joint manipulations of several of these components.

### Anti- and pro-synaptogenesis pathways and their interactions

The reported inhibition of Medea (Med) by Hiw [59] and the differential activity of Jun prompted us to analyze the effect of Med transcription factor and its partner, Mothers-against-dpp (Mad/Mnt), in the context of synaptogenesis. Interestingly, both factors exhibited coherent anti-synaptogenesis phenotypes. That is, decrease of synapse number in the ↑ condition and increase of synapses in the ↓ condition (Fig 4B). Mad and Med are known for their role in preventing cell differentiation to promote proliferation [101]. Here, they seem to reveal an anti-synaptogenesis signaling pathway without proliferation effects, given that axon numbers in the NMJ remained normal in these genotypes. Since transcription factors with putative pro- and anti-synaptogenesis roles had been identified, we explored the joint effects of some of them. The apparently ineffective Jun↓ condition suppressed the Mad↓ phenotype (Fig 4C). This result is relevant because it proves that the Jun↓ construct is functional (see above). Likewise, the Fos↓ condition suppressed the Mad↓ phenotype (Fig 4C). Also, the combination of two factors that yield synapse increase separately, Jun↑ and Mad↓ (Fig 4A and 4B) produced a synapse increase greatly attenuated with respect to the separate factors (Fig 4D). These data represent evidence that AP-1, either as a Jun/Fos heterodimer or as independent components, and Mad functionally interact in the context of synaptogenesis in an antagonistic manner.

**Fig 5:**
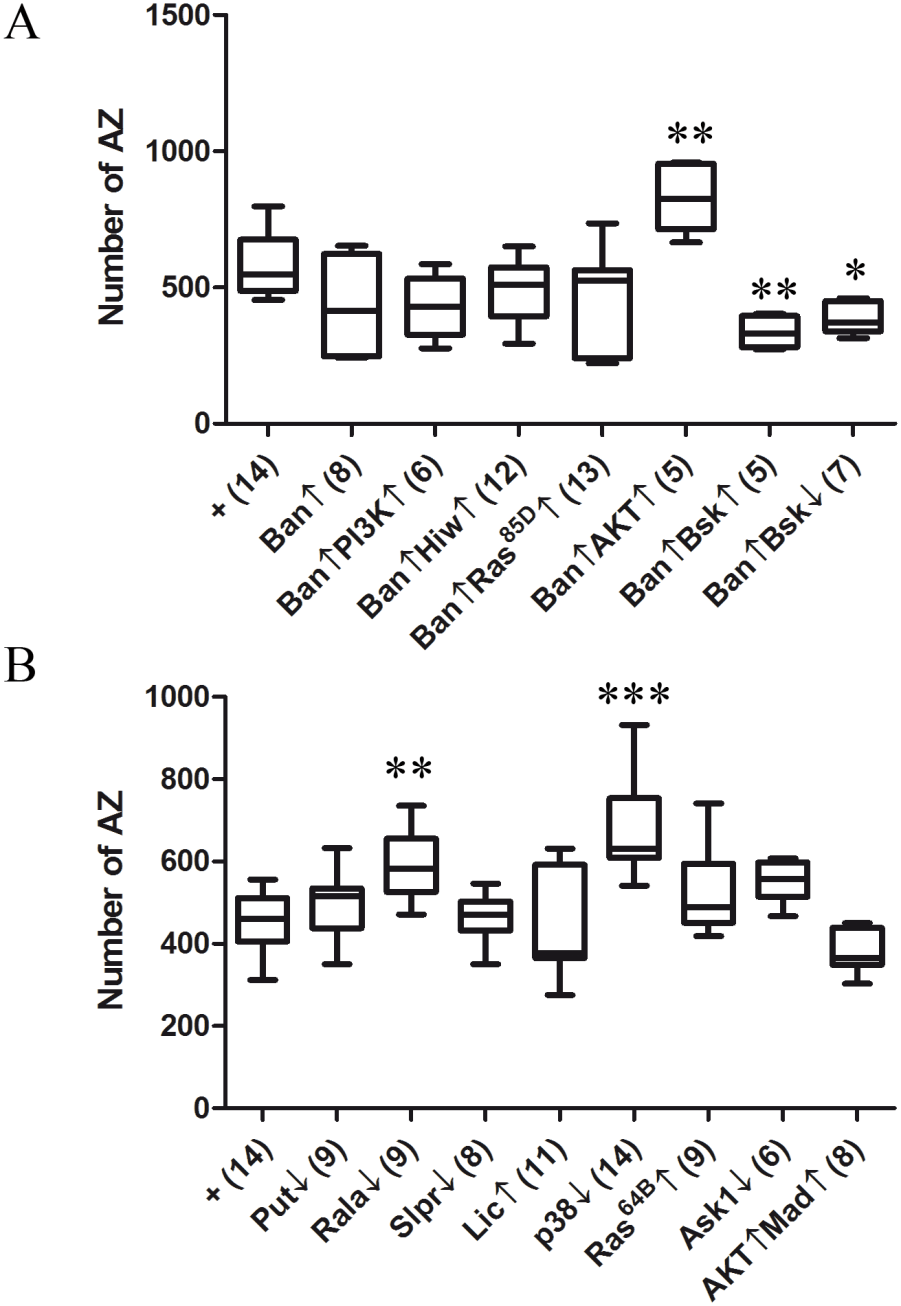
The anti-synaptogenesis pathway. **A**) The microRNA bantam shows a tendency towards synapse reduction which becomes evident by suppressing the synapse increase of PI3K↑, Hiw↑ and Bsk↑. By contrast, AKT escapes the repression by Ban since it maintains its synapse increase phenotype reported previously. Although below statistical significance, the pro-synaptogenesis tendency exhibited by Ras85D↑ seems also reverted by Ban. Also, the apparently ineffective Bsk↓ or the pro-synaptogenesis Bsk↑, both become anti-synaptogenic by Ban. These data justify the inclusion of Ban in the anti-synaptogenesis pathway and indicate additional targets for this microRNA. Genotypes: **+** = *D42-Gal4/+*. **Ban↑** = *D42-Gal4/UAS-bantam*. **Ban↑PI3K↑** = *UAS-PI3K/+; D42-Gal4/UAS-bantam*. **Ban↑Hiw↑** = *UAS-Hiw/+; D42-Gal4/UAS-bantam*. **Ban↑Ras85D↑** = *UAS-Ras85D/+; D42-Gal4/UAS-bantam*. **Ban↑AKT↑** = *D42-Gal4/UAS-bantam*,*UAS-Akt*. **Ban↑Bsk↑** = *UAS-Bsk/+; D42-Gal4/UAS-bantam*. **Ban↑Bsk↓** = *UAS-Bsk*^*DN*^*/+; +/+; D42-Gal4/UAS-bantam*. **B)** Assays of selected MAPK and GTPases candidates for the anti-synaptogenesis signaling. Genotypes: **+** = *D42-Gal4/+*. **Put↓** = *UAS-put*^*RNAi*^*/+; D42-Gal4/+*. **Rala↓** = *UAS-Rala*^*DN*^*/+; D42-Gal4/+*. **Slpr↓** = *D42-Gal4/UAS-Slpr*^*RNAi*^. **Lic↑** = *UAS-Lic/+; +/+; D42-Gal4/+*. **p38↓** = *UAS-p38a*^*RNAi*^*/+; D42-Gal4/+*. **Ras64B↑** = *UAS-Ras64B*^*V14*^*/+; D42-Gal4/+*. **Ask1↓** = *D42-Gal4/UAS-Ask1*^*RNAi*^. **AKT↑Mad↑** = *UAS-Akt/+; +/+; D42-Gal4/UAS-Mad*^*#2A2*^.

The canonical signaling pathway of Mad/Med includes the transcriptional co-regulator Yorkie and the microRNA bantam [102]. Thus, we tested bantam (Ban) in the context of synaptogenesis. The Ban↑ condition elicited a wide dispersion of synapse number values that render the tendency towards reduction as non-statistically significant. However, that condition suppressed the effects of PI3K↑, Hiw↑ and Ras85D↑ when jointly expressed (Fig 5A). In addition, the apparently ineffective Ban↑ transformed the phenotypes of Bsk↑ and Bsk↓ (Fig 3B) into a synapse reduction (Fig 5A). These data justified the allocation of Ban to the anti-synaptogenesis pathway and revealed its downregulation effects upon several elements of the pro-synaptogenesis pathway with the notable exception of Akt↑. The case of Akt is indicative that Ban does not target that gene and, in addition, that Akt has a substrate functionally downstream of Ban (see below). Thus, bantam should be included in the long list of microRNAs which contribute to synapse control [103].

The identification of transcriptional regulators with anti-synaptogenesis effects implies a corresponding signaling pathway that would counteract the pro-synaptogenesis pathway described hereto. Thus, we explored candidate genes that encode proteins akin to those constituting the pro-synaptogenesis signaling, MAPK and GTPases, mainly. The candidate genes were chosen based on previous reports of their functional interactions with *yorkie* (*yki*), a canonical member of the Mad/Medea transcriptional complex [104]. Out of the seven candidates tested, the two small GTPases Ras-like-a (Rala) and Ras64B (Ras64B), along with the two MAPKs Licorne (Lic) and p38a yielded synaptic phenotypes compatible with their adscription to the anti-synaptogenesis pathway (Fig 5B). Testing Licorne and p38a was justified because these two kinases share the same pathway in other cellular contexts [105, 106]. Here, p38 seems to contribute to the anti-synaptogenesis signaling while data on Licorne are too variable to draw a conclusion (Fig 5B). Concerning Rala, this neural GTPase plays a role in the activity-dependent changes at the postsynaptic side [107]. Thus, it seems coherent that, down-regulating it in the presynaptic side would increase synapse number. By contrast, the tyrosine kinase receptor punt (Put), the protein kinases Slipper (Slpr) and the apoptotic signaling-1 (Ask1/Pk92B) caused no effect (Fig 5B). The lack of effect of Ask1 represents another example of a context-dependent specificity for signaling. In human islet cells, Ask1 is functionally related to PI3K and JNK signaling [108], but it does not seems to be the case for synaptogenesis in fly neurons. Remarkably, all these anti-synaptogenesis components belong to the same functional classes (small GTPases, MAPK, etc.) as those of the pro-synaptogenesis pathway (see Discussion). The Yki transcriptional cofactor is known to receive signaling through the Hippo-Salvador-Warts pathway [109]. An alternative Hippo pathway has been reported that does not use Yki as a transcriptional effector and that, remarkably, mediates neuronal bouton formation [110]. This is another evidence of the strong context-dependence of signaling pathways.

**Fig 6:**
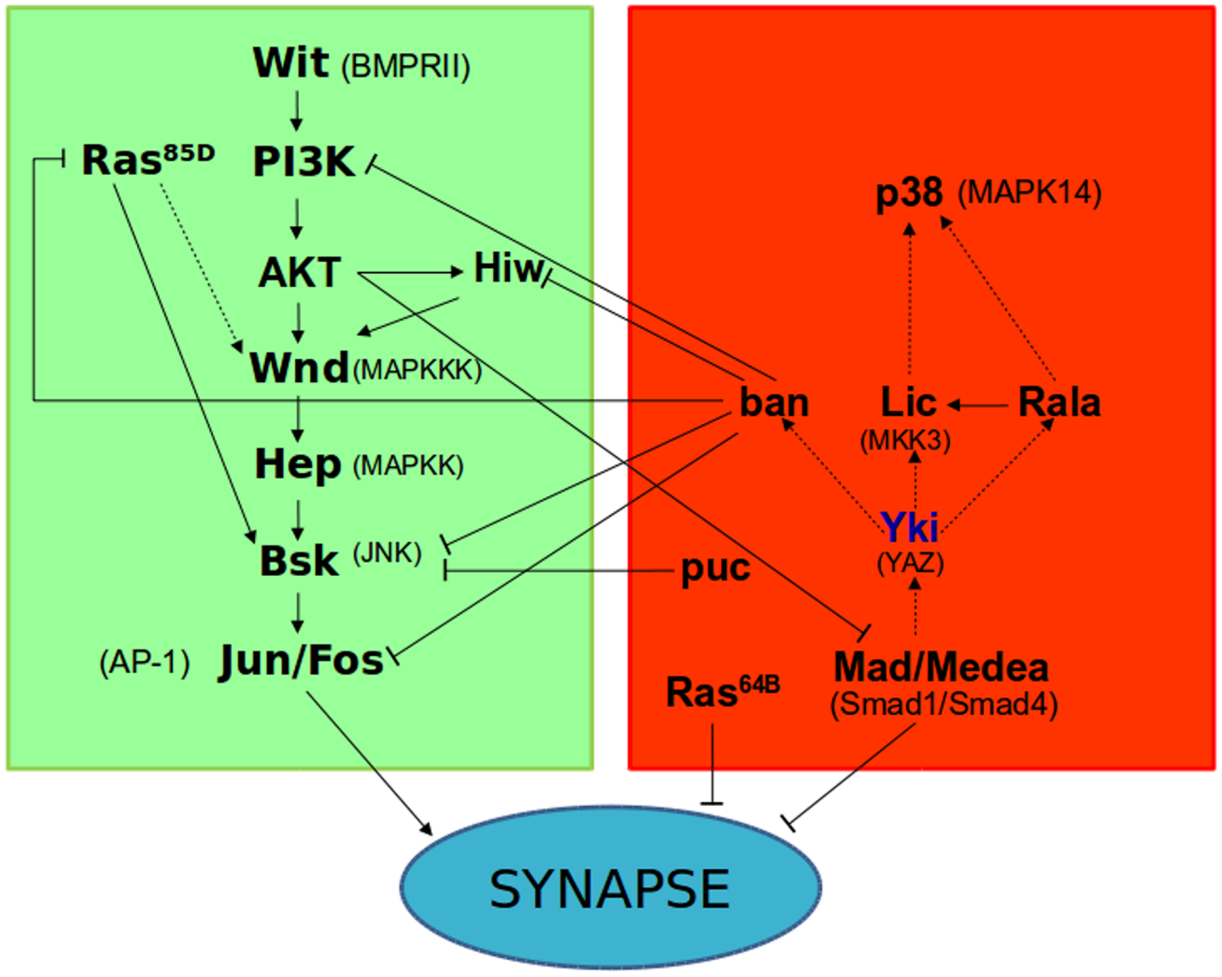
Summary diagram of antagonistic signaling pathways for synaptogenesis and their interactions. The pro-synaptogenesis pathway is shown in green while the anti-synaptogenesis one is shown in red. Arrows indicate activation and blunt lines indicate repression. Full lines correspond to interactions tested in this study and dotted lines refer to studies in other cellular contexts. The factor Yki was not included in this study and it is shown to illustrate interactions reported with the anti-synaptogenesis factors tested here. The vertebrate orthologues are shown between parentheses.

Finally, the substrate for Akt downstream from Ban that we hypothesized above was tested in the double genotype with Mad, the final effector of the anti-synaptogenesis pathway. Both elements together, Akt↑Mad↑, cancelled each other yielding a normal number of synapses (Fig 5B). This result is consistent with the observed lack of suppression of Akt↑ by Ban (Fig 5A) and suggests that Mad may be targeted by Akt in the context of synaptogenesis. The results in this report compose a scenario in which a delicate signaling balance between two antagonistic pathways determines the number of synapses that a neuron will establish under a particular functional status (Fig 6).

## Discussion

The epistasis assays have determined the *in vivo* functional links between PI3K and other previously known pro-synaptogenesis factors. Epistasis assays are based on the combined expression of two or more UAS constructs. Several double combinations in this study have produced a phenotype in spite of the apparent ineffectiveness of the single constructs. This type of results underscores the necessity to use epistasis assays in order to reveal functional interactions in vivo, hence, biologically relevant. In addition to the pro-synaptogenesis signaling, the study has revealed an anti-synaptogenesis pathway that composes a signaling equilibrium to determine the actual number of synapses. The magnitude of the synapse number changes elicited by the factors tested here are mostly within the range of 20–50%. Are these values significant to cause behavioral changes? Reductions in the order of 30% of excitatory or inhibitory synapses in adult *Drosophila* local olfactory interneurons transform perception of certain odorants from attraction to repulsion and vice versa [10]. In schizophrenia patients, a 16% loss of inhibitory synapses in the brain cortex has been reported [12]. In *Rhesus* monkeys, the pyramidal neurons in layer III of area 46 in dorsolateral prefrontal cortex show a 33% spine loss, and a significant reduction in learning task performance during normal aging [111]. Thus, it seems that behavior is rather sensitive to small changes in synapse number irrespective of the total brain mass.

The signaling interactions analyzed here were chosen because they were reported in other cellular systems and species previously. Some of these interactions have been confirmed (e.g.: Gbb/Wit), while others have proven ineffective in the context of synaptogenesis (e.g.: Ras85D/PI3K binding). Likely, the two signaling pathways, pro- and anti-synaptogenesis, are not the only ones relevant for synapse formation. For example, in spite of the null condition of the *gbb* and *wit* mutant alleles used here, the resulting synaptic phenotypes are far less extreme than expected if these two factors would be the only source of signaling for synaptogenesis. Although it could be argued that the incomplete absence of synapses in the mutant phenotypes could result from maternal perdurance, Wit is not part of the oocyte endowment while Gbb is. Three alternative possibilities may be considered, additional ligands for Wit, additional receptors for Gbb, and a combination of the previous two. Beyond the identity of these putative additional ligands and receptors, the stoichiometry between ligands and receptors may certainly be relevant. Actually, Gbb levels are titrated by Crimpy [68, 71]. An equivalent quantitative regulation could operate on Wit. The reported data on Wit illustrate already the diversity of the functional repertoire of this receptor. Wit can form heteromeric complexes with Thick veins (Tkv) or Saxophone (Sax) receptors to receive Dpp/BMP4 or Gbb/BMP7 as ligands [112, 113]. However, the same study also showed that Wit could dimerize with another receptor, Baboo, upon binding of Myoglianin to activate a different and antagonistic signaling pathway, TGFβ/activin-like [see also [114, 115]].

The Gbb/Wit/PI3K signaling analyzed here is likely not the only pro-synaptogenesis pathway in flies and vertebrates. The ligand wingless (Wg), member of the Wnt family, and the receptors Frizzled have been widely documented as relevant in neuromuscular junction development, albeit data on synapse number are scant [116, 117]. Interestingly, however, the downstream intermediaries can be as diverse as those mentioned above for Wit [118]. Although generally depicted as linear pathways, a more realistic image would be a network of cross-interacting signaling events whose in situ regulation and cellular compartmentalization remains fully unexplored.

The quantitative regulation of receptors is most relevant to understand their biological effects. In that context, is worth noting that Tkv levels are distinctly regulated from those of Wit and Sax through ubiquitination in the context of neurite growth [119]. On the other hand, although the receptor Wit is considered a RSTK type [120], the functional link with PI3K is a feature usually associated to the RTK type instead. The link of Wit with a kinase has a precedent with LIMK1 that binds to, and is functionally downstream from, Wit in the context of synapse stabilization [90]. Thus, Wit should be considered a wide spectrum receptor in terms of its ligands, co-receptor partners and, consequently, signaling pathways elicited. Actually, the Wit amino acid sequence shows both, Tyr and Ser/Thr motifs justifying its initial classification as a “dual” type of receptor (http://flybase.org). In this report we have not determined if Wit heterodimerizes with other receptors, as canonical RSTKs do, or if it forms homodimers, as canonical RTKs do. However, the lack of synaptogenesis effects by the putative co-receptors, Tkv and Sax, and the phenotypic similarity with the manipulation of the standard RTK signaling effector Cic, leaves open the possibility that Wit could play RTK-like functions, at least in the context of synaptogenesis.

Consistent with the proposal of a dual mechanism for Wit, its activation seems to be a requirement to elicit two independent signaling steps, PI3K and Ras85D, that could reflect RTK and RSTK mechanisms, respectively. Both steps are independent because the mutated form of PI3K unable to bind Ras85D, PI3K^ΔRBD^, is as effective as the normal PI3K to elicit synaptogenesis. PI3K and Ras85D signaling, however, seem to converge on Bsk revealing a novel feature of this crossroad point. The activity level of Bsk is known to be critical in many signaling processes [121, 122]. The peculiarities of Bsk/JNK activity include its coordinated regulation by p38a and Slpr in the context of stress heat response without interference on the developmental context [123]. Another modulator, Puc, was described as a negative feed-back loop in the context of oxidative stress [124]. The Puc mediated loop is operative also for synaptogenesis, while that of p38a/Slpr is relevant for p38a only, as shown here. Further, Ras85D represents an additional regulator in the neural scenario. The triple regulation of Bsk/JNK by Ras85D, Puc and the MAPKs seems to stablish a narrow range of activity thresholds within which normal number of synapses is determined.

The concept of signaling thresholds is also unveiled in this study by the identification of another signaling pathway that opposes synapse formation. The pro- and anti-synaptogenesis pathways have similar constituents, including small GTPases, MAPKs and transcriptional effectors, Mad/Smad, which are canonical for RSTK receptors. The RSTK type II receptor Put, which can mediate diverse signaling pathways according to the co-receptor bound [125] can be discarded in either the pro- or the anti-synaptogenesis pathways. Thus, the main receptor for the anti-synaptogenesis pathway remains to be identified.

Concerning small GTPases, the pro-synaptogenesis pathway uses Ras85D while its counterpart uses the poorly studied Ras64B. The anti-synaptogenesis pathway includes an additional member of this family of enzymes, Rala. This small GTPase plays a role in the exocyst-mediated growth of the muscle membrane specialization that surrounds the synaptic bouton as a consequence of synapse activity [107]. That is, Rala can influence synapse physiology acting from the postsynaptic side. The experimental expression of a constitutively active form of Rala in the neuron does not seem to affect the overall synaptic terminal branching [107]. However, the null *ral* mutant shows reduced synapse branching and its vertebrate homolog is expressed in the central nervous system [126]. We found here that Rala under-expression in neurons yields an elevated number of synapses. Thus, it is likely that this small GTPase acts as a break to synaptogenesis, hence its inclusion in the antagonistic pathway.

Synaptogenesis and neuritogenesis are distinct processes since each one can be differentially affected by the same mutant (e.g.: Hiw). Both features, however, share some signals (e.g.: Wnd, Hep). This signaling overlap is akin to the case of axon specification versus spine formation for constituents of the apico/basal polarity complex Par3-6/aPKC [127]. These and other examples illustrated in this study underscore the need to discriminate between synapses and boutons. This study is focused on the cell autonomous signaling that takes place in the neuron. Non-cell autonomous signals (e.g.: originated in the glia or hemolymph circulating) have not been considered. The active role of glia in axon pruning and bouton number has been the subject of other studies [128, 129]. Considering the reported role of Hiw through the midline glia in the remodeling of the giant fiber interneuron [91] it is not unlikely that the glia-to-neuron signaling may share components with the neuron autonomous signaling addressed here.

The summary scheme (Fig 6) describes the scenario where two signaling pathways mutually regulate each other. Epistasis assays are the only experimental approach for *in vivo* studies of more than one signaling component, albeit this type of assay is only feasible in *Drosophila*. The corresponding protein acronyms in humans are shown in parenthesis in Fig 6. Thus, it is plausible that vertebrate synaptogenesis will be regulated by a similar antagonistic signaling.

The regulatory equilibrium as a mechanism to determine a biological parameter is the most relevant feature in this scenario for several reasons. First, because this type of mechanism can respond very fast to changes in the physiological status of the cell, and, second because it provides remarkable precision to the trait to be regulated, synapse number in this case [see [130]]. Although bi-stable regulatory mechanisms are known in other contexts, the case of synapse number may seem unexpected because the highly dynamic nature of synapse number has been recognized only recently [1–3, 70]. Consequently, a molecular signaling mechanism endowed with proper precision and time resolution must sustain this dynamic process. The balanced equilibrium uncovered here, although most likely still incomplete in terms of its components, offers such a mechanism.

## Supporting information

S1 Fig Synapse visualization. **A)** Co-localization of synapse markers. Active zones are labeled in red with nc82 antibody while postsynaptic densities are shown in green (anti-GluRIID). The motor neuron membrane is shown in blue by the anti-HRP antibody. Note the correspondence of nc82 and GluRIID spots. **B)** The overexpression of the transcriptional repressor *capicua* (Cic) reduces synapses to the same level as the *wit*^*A12*^*/wit*^*B11*^ mutant (see also main Fig 1C). The dependence on *capicua* and the signaling through PI3K suggest that Wit may act as a RTK rather than a Ser/Thr kinase receptor. **C)** Postsynaptic FGFR-like receptors. The heterozygous mutant conditions for muscle receptors Htl or Btl increase synapse number. This is consistent with a repressive role in the levels of Gbb and, thus, in the increased pro-synaptogenesis signaling (see also S2 Fig). Genotypes: **Control** = *D42-Gal4/+*. **Cic↑** = *D42-Gal4/UAS-cic*. **Htl↓** = *htl*^*AB42*^*/+; D42-Gal4/+*. **Btl↓** = *btl*^*dev1*^*/+; D42-Gal4/+*. Bar in **A** = 5 μm. Number of independent larvae analyzed is shown in parenthesis. A single NMJ from abdominal segment A3 was analyzed per larvae.
(TIF)

S2 Fig Transcriptional effects on receptors and ligand-encoding genes in the muscle. **A**) Based on q-PCR assays, Gbb expression is increased as a result of the haploinsufficiency of Htl receptor. By contrast, the haploinsufficiency of Btl receptor leads to the opposite effect illustrating their antagonistic regulation on Gbb expression. The up- or down-regulation of PI3K in neurons has no effect on Gbb transcription. This result indicates that PI3K does not alter *gbb* transcription in the neuron nor triggers a signaling that could have modified that transcription in the muscle (see main text). **B-D**) The neural manipulation of PI3K has no effect on the Htl ligands, Pyramus (Pyr) (**B**) and Thisbe (Ths) (**C**), or on the Btl ligand Branchless (Bnl) (**D**). Genotypes: **+** = *elav-Gal4/+*. **Htl↓** = *htl*^*AB42*^*/+*. **Btl↑** = *btl*^*dev1*^*/+*. **PI3K↑** = *UAS-PI3K/+; elav-Gal4/+*. **PI3K↓** = *UAS-PI3K*^*DN*^*/+*; *elav-Gal4/+*.
(TIF)

S3 Fig Synaptogenesis and neuritogenesis are two different processes. **A-D**) Representative images of NMJs. The number of active zones and the size of the motor neuron deviate in opposite directions in *Hiw↓*. Motor neuron branches are more extensive than control but have fewer synapses. Also, note the frequent location of synapses, nc82 puncta, outside of boutons, particularly in Hiw↓. See also S3 Table. Genotypes: **A** = *D42-Gal4/+*. **B** = *UAS-Wnd/+*; *D42-Gal4/+*. **C** = *UAS-Hep/+*; *D42-Gal4/+*. **D** = males *hiw*^*ND8*^*; +/+; D42-Gal4/+*. Bar in **A** = 20 μm.
(TIF)

S1 Table Receptors without effect in synaptogenesis. (DOCX)

S2 Table Number of synapses (raw data). (XLS)

S3 Table Number of boutons versus number of synapses. (DOC)

S1 Movie 3D reconstruction of a NMJ. (AVI)

## References

[1] De Roo M, Klauser P, Muller D. LTP promotes a selective long-term stabilization and clustering of dendritic spines. PLoS biology. 2008;6(9):e219 Epub 2008/09/16. doi: 10.1371/journal.pbio.0060219 18788894 10.1371/journal.pbio.0060219 PMC2531136

[2] Martin-Pena A, Acebes A, Rodriguez JR, Sorribes A, de Polavieja GG, Fernandez-Funez P, et al Age-independent synaptogenesis by phosphoinositide 3 kinase. The Journal of neuroscience: the official journal of the Society for Neuroscience. 2006;26(40):10199–208. Epub 2006/10/06. 17021175 10.1523/JNEUROSCI.1223-06.2006 PMC6674615

[3] Rasse TM, Fouquet W, Schmid A, Kittel RJ, Mertel S, Sigrist CB, et al Glutamate receptor dynamics organizing synapse formation in vivo. Nature neuroscience. 2005;8(7):898–905. Epub 2005/09/02. 16136672 10.1038/nn1484

[4] Gan WB, Kwon E, Feng G, Sanes JR, Lichtman JW. Synaptic dynamism measured over minutes to months: age-dependent decline in an autonomic ganglion. Nature neuroscience. 2003;6(9):956–60. Epub 2003/08/20. doi: 10.1038/nn1115 12925856 10.1038/nn1115

[5] Middei S, Ammassari-Teule M, Marie H. Synaptic plasticity under learning challenge. Neurobiology of learning and memory. 2014;115:108–15. Epub 2014/08/19. doi: 10.1016/j.nlm.2014.08.001 25132316 10.1016/j.nlm.2014.08.001

[6] Trachtenberg JT, Chen BE, Knott GW, Feng G, Sanes JR, Welker E, et al Long-term in vivo imaging of experience-dependent synaptic plasticity in adult cortex. Nature. 2002;420(6917):788–94. Epub 2002/12/20. doi: 10.1038/nature01273 12490942 10.1038/nature01273

[7] Appelbaum L, Wang G, Yokogawa T, Skariah GM, Smith SJ, Mourrain P, et al Circadian and homeostatic regulation of structural synaptic plasticity in hypocretin neurons. Neuron. 2010;68(1):87–98. Epub 2010/10/06. doi: 10.1016/j.neuron.2010.09.006 20920793 10.1016/j.neuron.2010.09.006 PMC2969179

[8] Ruiz S, Ferreiro MJ, Menhert KI, Casanova G, Olivera A, Cantera R. Rhythmic changes in synapse numbers in Drosophila melanogaster motor terminals. PloS one. 2013;8(6):e67161 Epub 2013/07/11. doi: 10.1371/journal.pone.0067161 23840613 10.1371/journal.pone.0067161 PMC3695982

[9] Acebes A, Devaud JM, Arnes M, Ferrus A. Central adaptation to odorants depends on PI3K levels in local interneurons of the antennal lobe. The Journal of neuroscience: the official journal of the Society for Neuroscience. 2012;32(2):417–22. Epub 2012/01/13. 22238078 10.1523/JNEUROSCI.2921-11.2012 PMC6621088

[10] Acebes A, Martin-Pena A, Chevalier V, Ferrus A. Synapse loss in olfactory local interneurons modifies perception. The Journal of neuroscience: the official journal of the Society for Neuroscience. 2011;31(8):2734–45. Epub 2011/03/19. 21414896 10.1523/JNEUROSCI.5046-10.2011 PMC6623785

[11] Bernardinelli Y, Nikonenko I, Muller D. Structural plasticity: mechanisms and contribution to developmental psychiatric disorders. Frontiers in neuroanatomy. 2014;8:123 Epub 2014/11/19. doi: 10.3389/fnana.2014.00123 25404897 10.3389/fnana.2014.00123 PMC4217507

[12] Benes FM, Berretta S. GABAergic interneurons: implications for understanding schizophrenia and bipolar disorder. Neuropsychopharmacology: official publication of the American College of Neuropsychopharmacology. 2001;25(1):1–27. Epub 2001/05/30. 11377916 10.1016/S0893-133X(01)00225-1

[13] Scheff SW, Price DA. Synaptic pathology in Alzheimer's disease: a review of ultrastructural studies. Neurobiology of aging. 2003;24(8):1029–46. Epub 2003/12/04. 14643375 10.1016/j.neurobiolaging.2003.08.002

[14] Selkoe DJ. Alzheimer's disease is a synaptic failure. Science. 2002;298(5594):789–91. Epub 2002/10/26. doi: 10.1126/science.1074069 12399581 10.1126/science.1074069

[15] Ballard SL, Miller DL, Ganetzky B. Retrograde neurotrophin signaling through Tollo regulates synaptic growth in Drosophila. The Journal of cell biology. 2014;204(7):1157–72. Epub 2014/03/26. doi: 10.1083/jcb.201308115 24662564 10.1083/jcb.201308115 PMC3971753

[16] Baydyuk M, Wu XS, He L, Wu LG. Brain-derived neurotrophic factor inhibits calcium channel activation, exocytosis, and endocytosis at a central nerve terminal. The Journal of neuroscience: the official journal of the Society for Neuroscience. 2015;35(11):4676–82. Epub 2015/03/20. 25788684 10.1523/JNEUROSCI.2695-14.2015 PMC4363393

[17] Chen X, Ganetzky B. A neuropeptide signaling pathway regulates synaptic growth in Drosophila. The Journal of cell biology. 2012;196(4):529–43. Epub 2012/02/15. doi: 10.1083/jcb.201109044 22331845 10.1083/jcb.201109044 PMC3283997

[18] Chater TE, Goda Y. The role of AMPA receptors in postsynaptic mechanisms of synaptic plasticity. Frontiers in cellular neuroscience. 2014;8:401 Epub 2014/12/17. doi: 10.3389/fncel.2014.00401 25505875 10.3389/fncel.2014.00401 PMC4245900

[19] Chazeau A, Garcia M, Czondor K, Perrais D, Tessier B, Giannone G, et al Mechanical coupling between transsynaptic N-cadherin adhesions and actin flow stabilizes dendritic spines. Molecular biology of the cell. 2015;26(5):859–73. Epub 2015/01/09. doi: 10.1091/mbc.E14-06-1086 25568337 10.1091/mbc.E14-06-1086 PMC4342023

[20] Bayer KU, LeBel E, McDonald GL, O'Leary H, Schulman H, De Koninck P. Transition from reversible to persistent binding of CaMKII to postsynaptic sites and NR2B. The Journal of neuroscience: the official journal of the Society for Neuroscience. 2006;26(4):1164–74. Epub 2006/01/27. 16436603 10.1523/JNEUROSCI.3116-05.2006 PMC2890238

[21] Gleason MR, Higashijima S, Dallman J, Liu K, Mandel G, Fetcho JR. Translocation of CaM kinase II to synaptic sites in vivo. Nature neuroscience. 2003;6(3):217–8. Epub 2003/02/04. doi: 10.1038/nn1011 12563265 10.1038/nn1011

[22] Sturgill JF, Steiner P, Czervionke BL, Sabatini BL. Distinct domains within PSD-95 mediate synaptic incorporation, stabilization, and activity-dependent trafficking. The Journal of neuroscience: the official journal of the Society for Neuroscience. 2009;29(41):12845–54. Epub 2009/10/16. 19828799 10.1523/JNEUROSCI.1841-09.2009 PMC2787089

[23] Flores CE, Mendez P. Shaping inhibition: activity dependent structural plasticity of GABAergic synapses. Frontiers in cellular neuroscience. 2014;8:327 Epub 2014/11/12. doi: 10.3389/fncel.2014.00327 25386117 10.3389/fncel.2014.00327 PMC4209871

[24] Vonhoff F, Kuehn C, Blumenstock S, Sanyal S, Duch C. Temporal coherency between receptor expression, neural activity and AP-1-dependent transcription regulates Drosophila motoneuron dendrite development. Development. 2013;140(3):606–16. Epub 2013/01/08. doi: 10.1242/dev.089235 23293292 10.1242/dev.089235 PMC3561790

[25] Kordylewski L. Morphology of motor end-plate and its size relation to the muscle fibre size in the amphibian submandibular muscle. Zeitschrift fur mikroskopisch-anatomische Forschung. 1979;93(6):1038–50. Epub 1979/01/01. 317619

[26] Lewis ER, Cotman CW. Factors specifying the development of synapse number in the rat dentate gyrus: effects of partial target loss. Brain research. 1980;191(1):35–52. Epub 1980/06/02. 7378759 10.1016/0006-8993(80)90313-3

[27] Bailey CH, Kandel ER. Structural changes accompanying memory storage. Annual review of physiology. 1993;55:397–426. Epub 1993/01/01. doi: 10.1146/annurev.ph.55.030193.002145 8466181 10.1146/annurev.ph.55.030193.002145

[28] Ataman B, Ashley J, Gorczyca M, Ramachandran P, Fouquet W, Sigrist SJ, et al Rapid activity-dependent modifications in synaptic structure and function require bidirectional Wnt signaling. Neuron. 2008;57(5):705–18. Epub 2008/03/18. doi: 10.1016/j.neuron.2008.01.026 18341991 10.1016/j.neuron.2008.01.026 PMC2435264

[29] Nagaoka T, Ohashi R, Inutsuka A, Sakai S, Fujisawa N, Yokoyama M, et al The Wnt/planar cell polarity pathway component Vangl2 induces synapse formation through direct control of N-cadherin. Cell reports. 2014;6(5):916–27. Epub 2014/03/04. doi: 10.1016/j.celrep.2014.01.044 24582966 10.1016/j.celrep.2014.01.044

[30] Darabid H, Perez-Gonzalez AP, Robitaille R. Neuromuscular synaptogenesis: coordinating partners with multiple functions. Nature reviews Neuroscience. 2014;15(11):703–18. Epub 2014/12/11. 25493308

[31] Cuesto G, Jordan-Alvarez S, Enriquez-Barreto L, Ferrus A, Morales M, Acebes A. GSK3beta inhibition promotes synaptogenesis in Drosophila and mammalian neurons. PloS one. 2015;10(3):e0118475 Epub 2015/03/13. doi: 10.1371/journal.pone.0118475 25764078 10.1371/journal.pone.0118475 PMC4357437

[32] Cuesto G, Enriquez-Barreto L, Carames C, Cantarero M, Gasull X, Sandi C, et al Phosphoinositide-3-kinase activation controls synaptogenesis and spinogenesis in hippocampal neurons. The Journal of neuroscience: the official journal of the Society for Neuroscience. 2011;31(8):2721–33. Epub 2011/03/19. 21414895 10.1523/JNEUROSCI.4477-10.2011 PMC6623769

[33] Wang SH, Celic I, Choi SY, Riccomagno M, Wang Q, Sun LO, et al Dlg5 regulates dendritic spine formation and synaptogenesis by controlling subcellular N-cadherin localization. The Journal of neuroscience: the official journal of the Society for Neuroscience. 2014;34(38):12745–61. Epub 2014/09/19. 25232112 10.1523/JNEUROSCI.1280-14.2014 PMC4166160

[34] Ng SS, Mahmoudi T, Danenberg E, Bejaoui I, de Lau W, Korswagen HC, et al Phosphatidylinositol 3-kinase signaling does not activate the wnt cascade. The Journal of biological chemistry. 2009;284(51):35308–13. Epub 2009/10/24. doi: 10.1074/jbc.M109.078261 19850932 10.1074/jbc.M109.078261 PMC2790960

[35] Voskas D, Ling LS, Woodgett JR. Does GSK-3 provide a shortcut for PI3K activation of Wnt signalling? F1000 biology reports. 2010;2:82 Epub 2011/02/02. doi: 10.3410/B2-82 21283602 10.3410/B2-82 PMC3026644

[36] Cabodi S, Morello V, Masi A, Cicchi R, Broggio C, Distefano P, et al Convergence of integrins and EGF receptor signaling via PI3K/Akt/FoxO pathway in early gene Egr-1 expression. Journal of cellular physiology. 2009;218(2):294–303. Epub 2008/10/11. doi: 10.1002/jcp.21603 18844239 10.1002/jcp.21603

[37] Vanhaesebroeck B, Guillermet-Guibert J, Graupera M, Bilanges B. The emerging mechanisms of isoform-specific PI3K signalling. Nature reviews Molecular cell biology. 2010;11(5):329–41. Epub 2010/04/10. doi: 10.1038/nrm2882 20379207 10.1038/nrm2882

[38] Markus A, Zhong J, Snider WD. Raf and akt mediate distinct aspects of sensory axon growth. Neuron. 2002;35(1):65–76. Epub 2002/07/19. 12123609 10.1016/s0896-6273(02)00752-3

[39] Navarro AI, Rico B. Focal adhesion kinase function in neuronal development. Current opinion in neurobiology. 2014;27:89–95. Epub 2014/04/08. doi: 10.1016/j.conb.2014.03.002 24705242 10.1016/j.conb.2014.03.002

[40] Spilker C, Kreutz MR. RapGAPs in brain: multipurpose players in neuronal Rap signalling. The European journal of neuroscience. 2010;32(1):1–9. Epub 2010/06/26. doi: 10.1111/j.1460-9568.2010.07273.x 20576033 10.1111/j.1460-9568.2010.07273.x

[41] Acebes A, Ferrus A. Cellular and molecular features of axon collaterals and dendrites. Trends in neurosciences. 2000;23(11):557–65. Epub 2000/11/14. 11074265 10.1016/s0166-2236(00)01646-5

[42] Gallo G. The cytoskeletal and signaling mechanisms of axon collateral branching. Developmental neurobiology. 2011;71(3):201–20. Epub 2011/02/11. doi: 10.1002/dneu.20852 21308993 10.1002/dneu.20852

[43] Luo L, Liao YJ, Jan LY, Jan YN. Distinct morphogenetic functions of similar small GTPases: Drosophila Drac1 is involved in axonal outgrowth and myoblast fusion. Genes & development. 1994;8(15):1787–802. Epub 1994/08/01. 7958857 10.1101/gad.8.15.1787

[44] Parkes TL, Elia AJ, Dickinson D, Hilliker AJ, Phillips JP, Boulianne GL. Extension of Drosophila lifespan by overexpression of human SOD1 in motorneurons. Nature genetics. 1998;19(2):171–4. Epub 1998/06/10. doi: 10.1038/534 9620775 10.1038/534

[45] Orme MH, Alrubaie S, Bradley GL, Walker CD, Leevers SJ. Input from Ras is required for maximal PI(3)K signalling in Drosophila. Nature cell biology. 2006;8(11):1298–302. Epub 2006/10/17. doi: 10.1038/ncb1493 17041587 10.1038/ncb1493

[46] Riesgo-Escovar JR, Jenni M, Fritz A, Hafen E. The Drosophila Jun-N-terminal kinase is required for cell morphogenesis but not for DJun-dependent cell fate specification in the eye. Genes & development. 1996;10(21):2759–68. Epub 1996/11/01. 8946916 10.1101/gad.10.21.2759

[47] Rallis A, Moore C, Ng J. Signal strength and signal duration define two distinct aspects of JNK-regulated axon stability. Developmental biology. 2010;339(1):65–77. Epub 2009/12/29. doi: 10.1016/j.ydbio.2009.12.016 20035736 10.1016/j.ydbio.2009.12.016 PMC2845820

[48] Michelson AM, Gisselbrecht S, Buff E, Skeath JB. Heartbroken is a specific downstream mediator of FGF receptor signalling in Drosophila. Development. 1998;125(22):4379–89. Epub 1998/10/21. 9778498 10.1242/dev.125.22.4379

[49] Etter PD, Narayanan R, Navratilova Z, Patel C, Bohmann D, Jasper H, et al Synaptic and genomic responses to JNK and AP-1 signaling in Drosophila neurons. BMC neuroscience. 2005;6:39 Epub 2005/06/04. doi: 10.1186/1471-2202-6-39 15932641 10.1186/1471-2202-6-39 PMC1175850

[50] Collins CA, Wairkar YP, Johnson SL, DiAntonio A. Highwire restrains synaptic growth by attenuating a MAP kinase signal. Neuron. 2006;51(1):57–69. Epub 2006/07/04. doi: 10.1016/j.neuron.2006.05.026 16815332 10.1016/j.neuron.2006.05.026

[51] Asha H, Nagy I, Kovacs G, Stetson D, Ando I, Dearolf CR. Analysis of Ras-induced overproliferation in Drosophila hemocytes. Genetics. 2003;163(1):203–15. Epub 2003/02/15. 12586708 10.1093/genetics/163.1.203 PMC1462399

[52] Baonza A, de Celis JF, Garcia-Bellido A. Relationships between extramacrochaetae and Notch signalling in Drosophila wing development. Development. 2000;127(11):2383–93. Epub 2000/05/11. 10804180 10.1242/dev.127.11.2383

[53] Aberle H, Haghighi AP, Fetter RD, McCabe BD, Magalhaes TR, Goodman CS. wishful thinking encodes a BMP type II receptor that regulates synaptic growth in Drosophila. Neuron. 2002;33(4):545–58. Epub 2002/02/22. 11856529 10.1016/s0896-6273(02)00589-5

[54] Leevers SJ, Weinkove D, MacDougall LK, Hafen E, Waterfield MD. The Drosophila phosphoinositide 3-kinase Dp110 promotes cell growth. The EMBO journal. 1996;15(23):6584–94. Epub 1996/12/02. 8978685 PMC452483

[55] Wu C, Wairkar YP, Collins CA, DiAntonio A. Highwire function at the Drosophila neuromuscular junction: spatial, structural, and temporal requirements. The Journal of neuroscience: the official journal of the Society for Neuroscience. 2005;25(42):9557–66. Epub 2005/10/21. 16237161 10.1523/JNEUROSCI.2532-05.2005 PMC6725727

[56] Marques G, Bao H, Haerry TE, Shimell MJ, Duchek P, Zhang B, et al The Drosophila BMP type II receptor Wishful Thinking regulates neuromuscular synapse morphology and function. Neuron. 2002;33(4):529–43. Epub 2002/02/22. 11856528 10.1016/s0896-6273(02)00595-0

[57] Khalsa O, Yoon JW, Torres-Schumann S, Wharton KA. TGF-beta/BMP superfamily members, Gbb-60A and Dpp, cooperate to provide pattern information and establish cell identity in the Drosophila wing. Development. 1998;125(14):2723–34. Epub 1998/06/24. 9636086 10.1242/dev.125.14.2723

[58] Wharton KA, Cook JM, Torres-Schumann S, de Castro K, Borod E, Phillips DA. Genetic analysis of the bone morphogenetic protein-related gene, gbb, identifies multiple requirements during Drosophila development. Genetics. 1999;152(2):629–40. Epub 1999/06/03. 10353905 10.1093/genetics/152.2.629 PMC1460618

[59] McCabe BD, Marques G, Haghighi AP, Fetter RD, Crotty ML, Haerry TE, et al The BMP homolog Gbb provides a retrograde signal that regulates synaptic growth at the Drosophila neuromuscular junction. Neuron. 2003;39(2):241–54. Epub 2003/07/23. 12873382 10.1016/s0896-6273(03)00426-4

[60] Wan HI, DiAntonio A, Fetter RD, Bergstrom K, Strauss R, Goodman CS. Highwire regulates synaptic growth in Drosophila. Neuron. 2000;26(2):313–29. Epub 2000/06/06. 10839352 10.1016/s0896-6273(00)81166-6

[61] Schuster CM, Davis GW, Fetter RD, Goodman CS. Genetic dissection of structural and functional components of synaptic plasticity. I. Fasciclin II controls synaptic stabilization and growth. Neuron. 1996;17(4):641–54. Epub 1996/10/01. 8893022 10.1016/s0896-6273(00)80197-x

[62] Hipfner DR, Weigmann K, Cohen SM. The bantam gene regulates Drosophila growth. Genetics. 2002;161(4):1527–37. Epub 2002/08/28. 12196398 10.1093/genetics/161.4.1527 PMC1462212

[63] Budnik VE, Ruiz-Canada CE. The Fly Neuromuscular Junction: Structure and Function: ACADEMIC PRESS; 2006 10 2006.

[64] Brand AH, Perrimon N. Targeted gene expression as a means of altering cell fates and generating dominant phenotypes. Development. 1993;118(2):401–15. Epub 1993/06/01. 8223268 10.1242/dev.118.2.401

[65] Wagh DA, Rasse TM, Asan E, Hofbauer A, Schwenkert I, Durrbeck H, et al Bruchpilot, a protein with homology to ELKS/CAST, is required for structural integrity and function of synaptic active zones in Drosophila. Neuron. 2006;49(6):833–44. Epub 2006/03/18. doi: 10.1016/j.neuron.2006.02.008 16543132 10.1016/j.neuron.2006.02.008

[66] Bao H, Daniels RW, MacLeod GT, Charlton MP, Atwood HL, Zhang B. AP180 maintains the distribution of synaptic and vesicle proteins in the nerve terminal and indirectly regulates the efficacy of Ca2+-triggered exocytosis. J Neurophysiol. 2005;94(3):1888–903. doi: 10.1152/jn.00080.2005 15888532 10.1152/jn.00080.2005

[67] Fouquet W, Owald D, Wichmann C, Mertel S, Depner H, Dyba M, et al Maturation of active zone assembly by Drosophila Bruchpilot. The Journal of cell biology. 2009;186(1):129–45. Epub 2009/07/15. doi: 10.1083/jcb.200812150 19596851 10.1083/jcb.200812150 PMC2712991

[68] Guo X, Macleod GT, Wellington A, Hu F, Panchumarthi S, Schoenfield M, et al The GTPase dMiro is required for axonal transport of mitochondria to Drosophila synapses. Neuron. 2005;47(3):379–93. doi: 10.1016/j.neuron.2005.06.027 16055062 10.1016/j.neuron.2005.06.027

[69] Hamanaka Y, Meinertzhagen IA. Immunocytochemical localization of synaptic proteins to photoreceptor synapses of Drosophila melanogaster. The Journal of comparative neurology. 2010;518(7):1133–55. Epub 2010/02/04. doi: 10.1002/cne.22268 20127822 10.1002/cne.22268 PMC4029604

[70] Jordan-Alvarez S, Fouquet W, Sigrist SJ, Acebes A. Presynaptic PI3K activity triggers the formation of glutamate receptors at neuromuscular terminals of Drosophila. Journal of cell science. 2012;125(Pt 15):3621–9. Epub 2012/04/17. doi: 10.1242/jcs.102806 22505608 10.1242/jcs.102806

[71] Peled ES, Newman ZL, Isacoff EY. Evoked and spontaneous transmission favored by distinct sets of synapses. Current biology: CB. 2014;24(5):484–93. Epub 2014/02/25. doi: 10.1016/j.cub.2014.01.022 24560571 10.1016/j.cub.2014.01.022 PMC4017949

[72] Marques G. Morphogens and synaptogenesis in Drosophila. Journal of neurobiology. 2005;64(4):417–34. Epub 2005/07/26. doi: 10.1002/neu.20165 16041756 10.1002/neu.20165

[73] James RE, Broihier HT. Crimpy inhibits the BMP homolog Gbb in motoneurons to enable proper growth control at the Drosophila neuromuscular junction. Development. 2011;138(15):3273–86. Epub 2011/07/14. doi: 10.1242/dev.066142 21750037 10.1242/dev.066142 PMC3133917

[74] Banerjee S, Venkatesan A, Bhat MA. Neurexin, Neuroligin and Wishful Thinking coordinate synaptic cytoarchitecture and growth at neuromuscular junctions. Molecular and cellular neurosciences. 2016;78:9–24. Epub 2016/11/14. doi: 10.1016/j.mcn.2016.11.004 27838296 10.1016/j.mcn.2016.11.004 PMC5219945

[75] Kim NC, Marques G. Identification of downstream targets of the bone morphogenetic protein pathway in the Drosophila nervous system. Developmental dynamics: an official publication of the American Association of Anatomists. 2010;239(9):2413–25. Epub 2010/07/24. 20652954 10.1002/dvdy.22368

[76] James RE, Hoover KM, Bulgari D, McLaughlin CN, Wilson CG, Wharton KA, et al Crimpy enables discrimination of presynaptic and postsynaptic pools of a BMP at the Drosophila neuromuscular junction. Developmental cell. 2014;31(5):586–98. Epub 2014/12/03. doi: 10.1016/j.devcel.2014.10.006 25453556 10.1016/j.devcel.2014.10.006 PMC4283799

[77] Jimenez G, Shvartsman SY, Paroush Z. The Capicua repressor—a general sensor of RTK signaling in development and disease. Journal of cell science. 2012;125(Pt 6):1383–91. Epub 2012/04/25. doi: 10.1242/jcs.092965 22526417 10.1242/jcs.092965 PMC3336375

[78] Reichman-Fried M, Shilo BZ. Breathless, a Drosophila FGF receptor homolog, is required for the onset of tracheal cell migration and tracheole formation. Mechanisms of development. 1995;52(2–3):265–73. Epub 1995/08/01. 8541215 10.1016/0925-4773(95)00407-r

[79] Shishido E, Higashijima S, Emori Y, Saigo K. Two FGF-receptor homologues of Drosophila: one is expressed in mesodermal primordium in early embryos. Development. 1993;117(2):751–61. Epub 1993/02/01. 8330538 10.1242/dev.117.2.751

[80] Emori Y, Saigo K. Distinct expression of two Drosophila homologs of fibroblast growth factor receptors in imaginal discs. FEBS letters. 1993;332(1–2):111–4. Epub 1993/10/11. 8405423 10.1016/0014-5793(93)80494-f

[81] Gryzik T, Muller HA. FGF8-like1 and FGF8-like2 encode putative ligands of the FGF receptor Htl and are required for mesoderm migration in the Drosophila gastrula. Current biology: CB. 2004;14(8):659–67. Epub 2004/04/16. doi: 10.1016/j.cub.2004.03.058 15084280 10.1016/j.cub.2004.03.058

[82] Reichman-Fried M, Dickson B, Hafen E, Shilo BZ. Elucidation of the role of breathless, a Drosophila FGF receptor homolog, in tracheal cell migration. Genes & development. 1994;8(4):428–39. Epub 1994/02/15. 8125257 10.1101/gad.8.4.428

[83] Stathopoulos A, Tam B, Ronshaugen M, Frasch M, Levine M. pyramus and thisbe: FGF genes that pattern the mesoderm of Drosophila embryos. Genes & development. 2004;18(6):687–99. Epub 2004/04/13. 15075295 10.1101/gad.1166404 PMC387243

[84] Kadam S, McMahon A, Tzou P, Stathopoulos A. FGF ligands in Drosophila have distinct activities required to support cell migration and differentiation. Development. 2009;136(5):739–47. Epub 2009/01/23. doi: 10.1242/dev.027904 19158183 10.1242/dev.027904 PMC2685942

[85] Berke B, Wittnam J, McNeill E, Van Vactor DL, Keshishian H. Retrograde BMP signaling at the synapse: a permissive signal for synapse maturation and activity-dependent plasticity. The Journal of neuroscience: the official journal of the Society for Neuroscience. 2013;33(45):17937–50. Epub 2013/11/08. 24198381 10.1523/JNEUROSCI.6075-11.2013 PMC3818560

[86] Seeger G, Gartner U, Arendt T. Transgenic activation of Ras in neurons increases synapse formation in mouse neocortex. J Neural Transm. 2005;112(6):751–61. Epub 2004/10/14. doi: 10.1007/s00702-004-0226-8 15480849 10.1007/s00702-004-0226-8

[87] Castellano E, Downward J. RAS Interaction with PI3K: More Than Just Another Effector Pathway. Genes & cancer. 2011;2(3):261–74. Epub 2011/07/23. 21779497 10.1177/1947601911408079 PMC3128635

[88] Schaefer AM, Hadwiger GD, Nonet ML. rpm-1, a conserved neuronal gene that regulates targeting and synaptogenesis in C. elegans. Neuron. 2000;26(2):345–56. Epub 2000/06/06. 10839354 10.1016/s0896-6273(00)81168-x

[89] Raftery LA, Sutherland DJ. TGF-beta family signal transduction in Drosophila development: from Mad to Smads. Developmental biology. 1999;210(2):251–68. Epub 1999/06/08. doi: 10.1006/dbio.1999.9282 10357889 10.1006/dbio.1999.9282

[90] Eaton BA, Davis GW. LIM Kinase1 controls synaptic stability downstream of the type II BMP receptor. Neuron. 2005;47(5):695–708. Epub 2005/09/01. doi: 10.1016/j.neuron.2005.08.010 16129399 10.1016/j.neuron.2005.08.010

[91] Borgen M, Rowland K, Boerner J, Lloyd B, Khan A, Murphey R. Axon Termination, Pruning, and Synaptogenesis in the Giant Fiber System of Drosophila melanogaster Is Promoted by Highwire. Genetics. 2017;205(3):1229–45. Epub 2017/01/20. doi: 10.1534/genetics.116.197343 28100586 10.1534/genetics.116.197343 PMC5340335

[92] Haeusgen W, Herdegen T, Waetzig V. The bottleneck of JNK signaling: molecular and functional characteristics of MKK4 and MKK7. European journal of cell biology. 2011;90(6–7):536–44. Epub 2011/02/22. doi: 10.1016/j.ejcb.2010.11.008 21333379 10.1016/j.ejcb.2010.11.008

[93] Geuking P, Narasimamurthy R, Lemaitre B, Basler K, Leulier F. A non-redundant role for Drosophila Mkk4 and hemipterous/Mkk7 in TAK1-mediated activation of JNK. PloS one. 2009;4(11):e7709 Epub 2009/11/06. doi: 10.1371/journal.pone.0007709 19888449 10.1371/journal.pone.0007709 PMC2766050

[94] Martin-Blanco E, Gampel A, Ring J, Virdee K, Kirov N, Tolkovsky AM, et al puckered encodes a phosphatase that mediates a feedback loop regulating JNK activity during dorsal closure in Drosophila. Genes & development. 1998;12(4):557–70. Epub 1998/03/21. 9472024 10.1101/gad.12.4.557 PMC316530

[95] Ciapponi L, Bohmann D. An essential function of AP-1 heterodimers in Drosophila development. Mechanisms of development. 2002;115(1–2):35–40. Epub 2002/06/07. 12049765 10.1016/s0925-4773(02)00093-x

[96] Collins CA, DiAntonio A. Coordinating synaptic growth without being a nervous wreck. Neuron. 2004;41(4):489–91. Epub 2004/02/26. 14980197 10.1016/s0896-6273(04)00078-9

[97] Gass P, Fleischmann A, Hvalby O, Jensen V, Zacher C, Strekalova T, et al Mice with a fra-1 knock-in into the c-fos locus show impaired spatial but regular contextual learning and normal LTP. Brain research Molecular brain research. 2004;130(1–2):16–22. Epub 2004/11/03. doi: 10.1016/j.molbrainres.2004.07.004 15519672 10.1016/j.molbrainres.2004.07.004

[98] Sanyal S, Sandstrom DJ, Hoeffer CA, Ramaswami M. AP-1 functions upstream of CREB to control synaptic plasticity in Drosophila. Nature. 2002;416(6883):870–4. Epub 2002/04/27. doi: 10.1038/416870a 11976688 10.1038/416870a

[99] Dobens LL, Martin-Blanco E, Martinez-Arias A, Kafatos FC, Raftery LA. Drosophila puckered regulates Fos/Jun levels during follicle cell morphogenesis. Development. 2001;128(10):1845–56. Epub 2001/04/20. 11311164 10.1242/dev.128.10.1845

[100] Szuts D, Bienz M. LexA chimeras reveal the function of Drosophila Fos as a context-dependent transcriptional activator. Proceedings of the National Academy of Sciences of the United States of America. 2000;97(10):5351–6. Epub 2000/05/11. 10805795 10.1073/pnas.97.10.5351 PMC25832

[101] Brummel T, Abdollah S, Haerry TE, Shimell MJ, Merriam J, Raftery L, et al The Drosophila activin receptor baboon signals through dSmad2 and controls cell proliferation but not patterning during larval development. Genes & development. 1999;13(1):98–111. Epub 1999/01/14. 9887103 10.1101/gad.13.1.98 PMC316373

[102] Oh H, Irvine KD. Cooperative regulation of growth by Yorkie and Mad through bantam. Developmental cell. 2011;20(1):109–22. Epub 2011/01/18. doi: 10.1016/j.devcel.2010.12.002 21238929 10.1016/j.devcel.2010.12.002 PMC3033745

[103] McNeill E, Van Vactor D. MicroRNAs shape the neuronal landscape. Neuron. 2012;75(3):363–79. Epub 2012/08/14. doi: 10.1016/j.neuron.2012.07.005 22884321 10.1016/j.neuron.2012.07.005 PMC3441179

[104] Kwon Y, Vinayagam A, Sun X, Dephoure N, Gygi SP, Hong P, et al The Hippo signaling pathway interactome. Science. 2013;342(6159):737–40. Epub 2013/10/12. doi: 10.1126/science.1243971 24114784 10.1126/science.1243971 PMC3951131

[105] Sekine Y, Takagahara S, Hatanaka R, Watanabe T, Oguchi H, Noguchi T, et al p38 MAPKs regulate the expression of genes in the dopamine synthesis pathway through phosphorylation of NR4A nuclear receptors. Journal of cell science. 2011;124(Pt 17):3006–16. Epub 2011/09/01. doi: 10.1242/jcs.085902 21878507 10.1242/jcs.085902

[106] Han ZS, Enslen H, Hu X, Meng X, Wu IH, Barrett T, et al A conserved p38 mitogen-activated protein kinase pathway regulates Drosophila immunity gene expression. Molecular and cellular biology. 1998;18(6):3527–39. Epub 1998/06/20. 9584193 10.1128/mcb.18.6.3527 PMC108934

[107] Teodoro RO, Pekkurnaz G, Nasser A, Higashi-Kovtun ME, Balakireva M, McLachlan IG, et al Ral mediates activity-dependent growth of postsynaptic membranes via recruitment of the exocyst. The EMBO journal. 2013;32(14):2039–55. Epub 2013/07/03. doi: 10.1038/emboj.2013.147 23812009 10.1038/emboj.2013.147 PMC3715865

[108] Aikin R, Maysinger D, Rosenberg L. Cross-talk between phosphatidylinositol 3-kinase/AKT and c-jun NH2-terminal kinase mediates survival of isolated human islets. Endocrinology. 2004;145(10):4522–31. Epub 2004/07/10. doi: 10.1210/en.2004-0488 15242986 10.1210/en.2004-0488

[109] Pfleger CM. The Hippo Pathway: A Master Regulatory Network Important in Development and Dysregulated in Disease. Current topics in developmental biology. 2017;123:181–228. Epub 2017/02/27. doi: 10.1016/bs.ctdb.2016.12.001 28236967 10.1016/bs.ctdb.2016.12.001

[110] Sakuma C, Saito Y, Umehara T, Kamimura K, Maeda N, Mosca TJ, et al The Strip-Hippo Pathway Regulates Synaptic Terminal Formation by Modulating Actin Organization at the Drosophila Neuromuscular Synapses. Cell reports. 2016;16(9):2289–97. Epub 2016/08/23. doi: 10.1016/j.celrep.2016.07.066 27545887 10.1016/j.celrep.2016.07.066 PMC5023852

[111] Dumitriu D, Hao J, Hara Y, Kaufmann J, Janssen WG, Lou W, et al Selective changes in thin spine density and morphology in monkey prefrontal cortex correlate with aging-related cognitive impairment. The Journal of neuroscience: the official journal of the Society for Neuroscience. 2010;30(22):7507–15. Epub 2010/06/04. 20519525 10.1523/JNEUROSCI.6410-09.2010 PMC2892969

[112] Lee-Hoeflich ST, Zhao X, Mehra A, Attisano L. The Drosophila type II receptor, Wishful thinking, binds BMP and myoglianin to activate multiple TGFbeta family signaling pathways. FEBS letters. 2005;579(21):4615–21. Epub 2005/08/16. doi: 10.1016/j.febslet.2005.06.088 16098524 10.1016/j.febslet.2005.06.088

[113] Bangi E, Wharton K. Dual function of the Drosophila Alk1/Alk2 ortholog Saxophone shapes the Bmp activity gradient in the wing imaginal disc. Development. 2006;133(17):3295–303. Epub 2006/08/05. doi: 10.1242/dev.02513 16887821 10.1242/dev.02513

[114] Demontis F, Patel VK, Swindell WR, Perrimon N. Intertissue control of the nucleolus via a myokine-dependent longevity pathway. Cell reports. 2014;7(5):1481–94. Epub 2014/06/03. doi: 10.1016/j.celrep.2014.05.001 24882005 10.1016/j.celrep.2014.05.001 PMC4125979

[115] Ghosh AC, O'Connor MB. Systemic Activin signaling independently regulates sugar homeostasis, cellular metabolism, and pH balance in Drosophila melanogaster. Proceedings of the National Academy of Sciences of the United States of America. 2014;111(15):5729–34. Epub 2014/04/08. doi: 10.1073/pnas.1319116111 24706779 10.1073/pnas.1319116111 PMC3992655

[116] Koles K, Nunnari J, Korkut C, Barria R, Brewer C, Li Y, et al Mechanism of evenness interrupted (Evi)-exosome release at synaptic boutons. The Journal of biological chemistry. 2012;287(20):16820–34. Epub 2012/03/23. doi: 10.1074/jbc.M112.342667 22437826 10.1074/jbc.M112.342667 PMC3351299

[117] Korkut C, Ataman B, Ramachandran P, Ashley J, Barria R, Gherbesi N, et al Trans-synaptic transmission of vesicular Wnt signals through Evi/Wntless. Cell. 2009;139(2):393–404. Epub 2009/10/20. doi: 10.1016/j.cell.2009.07.051 19837038 10.1016/j.cell.2009.07.051 PMC2785045

[118] Messeant J, Ezan J, Delers P, Glebov K, Marchiol C, Lager F, et al Wnts contribute to neuromuscular junction formation through distinct signaling pathways. Development. 2017. Epub 2017/03/30. 10.1242/dev.146167 28348167

[119] Li W, Yao A, Zhi H, Kaur K, Zhu YC, Jia M, et al Angelman Syndrome Protein Ube3a Regulates Synaptic Growth and Endocytosis by Inhibiting BMP Signaling in Drosophila. PLoS genetics. 2016;12(5):e1006062 Epub 2016/05/28. doi: 10.1371/journal.pgen.1006062 27232889 10.1371/journal.pgen.1006062 PMC4883773

[120] Alexander PB, Wang XF. Resistance to receptor tyrosine kinase inhibition in cancer: molecular mechanisms and therapeutic strategies. Frontiers of medicine. 2015;9(2):134–8. Epub 2015/05/10. doi: 10.1007/s11684-015-0396-9 25957263 10.1007/s11684-015-0396-9 PMC4535791

[121] Zhou YY, Li Y, Jiang WQ, Zhou LF. MAPK/JNK signalling: a potential autophagy regulation pathway. Bioscience reports. 2015;35(3). Epub 2015/07/17. 10.1042/BSR20140141 PMC4613668 26182361

[122] Zhao HF, Wang J, Tony To SS. The phosphatidylinositol 3-kinase/Akt and c-Jun N-terminal kinase signaling in cancer: Alliance or contradiction? (Review). International journal of oncology. 2015;47(2):429–36. Epub 2015/06/18. doi: 10.3892/ijo.2015.3052 26082006 10.3892/ijo.2015.3052

[123] Gonda RL, Garlena RA, Stronach B. Drosophila heat shock response requires the JNK pathway and phosphorylation of mixed lineage kinase at a conserved serine-proline motif. PloS one. 2012;7(7):e42369 Epub 2012/08/01. doi: 10.1371/journal.pone.0042369 22848763 10.1371/journal.pone.0042369 PMC3407086

[124] Karkali K, Panayotou G. The Drosophila DUSP puckered is phosphorylated by JNK and p38 in response to arsenite-induced oxidative stress. Biochemical and biophysical research communications. 2012;418(2):301–6. Epub 2012/01/24. doi: 10.1016/j.bbrc.2012.01.015 22266315 10.1016/j.bbrc.2012.01.015

[125] Parker L, Ellis JE, Nguyen MQ, Arora K. The divergent TGF-beta ligand Dawdle utilizes an activin pathway to influence axon guidance in Drosophila. Development. 2006;133(24):4981–91. Epub 2006/11/23. doi: 10.1242/dev.02673 17119022 10.1242/dev.02673

[126] Lalli G. RalA and the exocyst complex influence neuronal polarity through PAR-3 and aPKC. Journal of cell science. 2009;122(Pt 10):1499–506. Epub 2009/04/23. doi: 10.1242/jcs.044339 19383721 10.1242/jcs.044339

[127] Zhang H, Macara IG. The PAR-6 polarity protein regulates dendritic spine morphogenesis through p190 RhoGAP and the Rho GTPase. Developmental cell. 2008;14(2):216–26. Epub 2008/02/13. doi: 10.1016/j.devcel.2007.11.020 18267090 10.1016/j.devcel.2007.11.020 PMC2768076

[128] Awasaki T, Ito K. Engulfing action of glial cells is required for programmed axon pruning during Drosophila metamorphosis. Current biology: CB. 2004;14(8):668–77. Epub 2004/04/16. doi: 10.1016/j.cub.2004.04.001 15084281 10.1016/j.cub.2004.04.001

[129] Fuentes-Medel Y, Logan MA, Ashley J, Ataman B, Budnik V, Freeman MR. Glia and muscle sculpt neuromuscular arbors by engulfing destabilized synaptic boutons and shed presynaptic debris. PLoS biology. 2009;7(8):e1000184 Epub 2009/08/27. doi: 10.1371/journal.pbio.1000184 19707574 10.1371/journal.pbio.1000184 PMC2724735

[130] Marucci L, Barton DA, Cantone I, Ricci MA, Cosma MP, Santini S, et al How to turn a genetic circuit into a synthetic tunable oscillator, or a bistable switch. PloS one. 2009;4(12):e8083 Epub 2009/12/10. doi: 10.1371/journal.pone.0008083 19997611 10.1371/journal.pone.0008083 PMC2784219

