# Supplementary figures and images for: The equilibrium between antagonistic signaling pathways determines the number of synapses in Drosophila
Source: PLoS One. 2017 Sep 11;12(9):e0184238. doi: 10.1371/journal.pone.0184238 (PMC5593197; doi:10.1371/journal.pone.0184238)

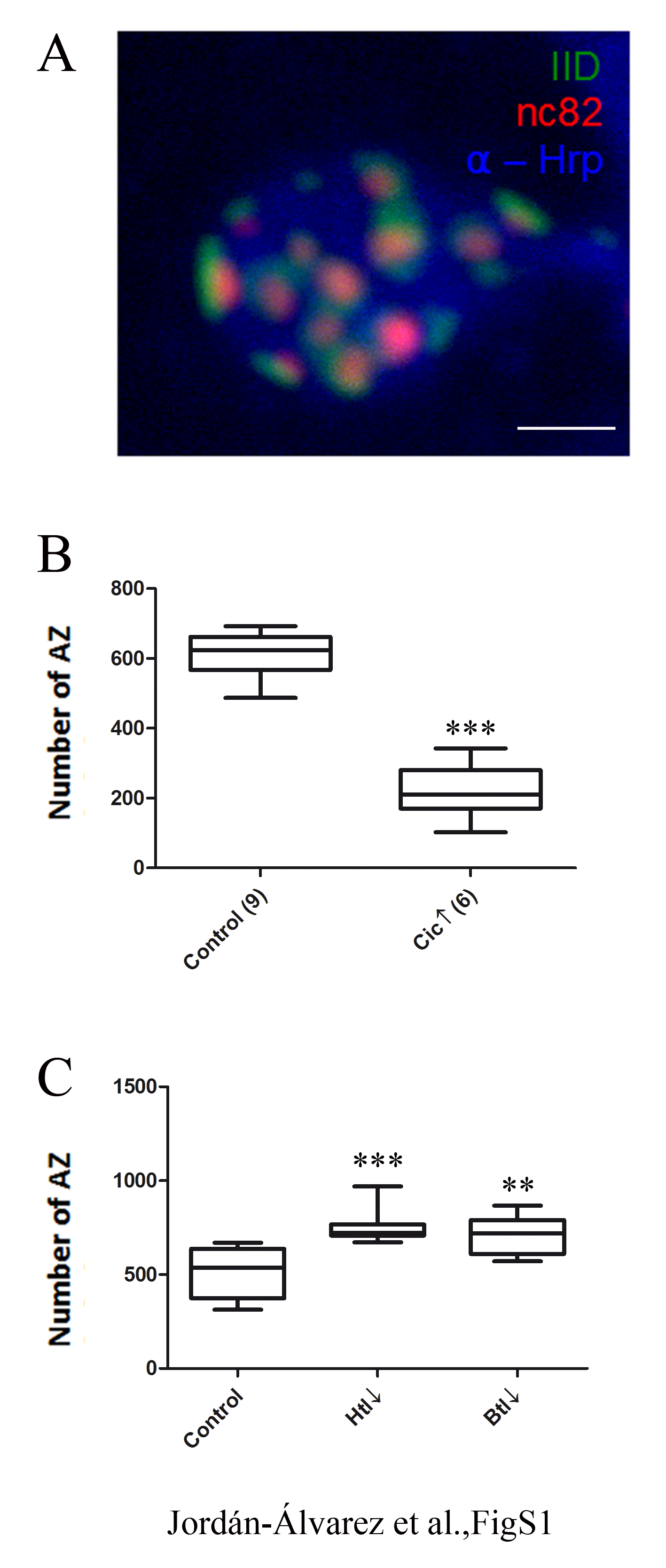

Supplement: S1 Fig — A) Co-localization of synapse markers. Active zones are labeled in red with nc82 antibody while postsynaptic densities are shown in green (anti-GluRIID). The motor neuron membrane is shown in blue by the anti-HRP antibody. Note the correspondence of nc82 and GluRIID spots. B) The overexpression of the transcriptional repressor capicua (Cic) reduces synapses to the same level as the witA12/witB11 mutant (see also main Fig 1C). The dependence on capicua and the signaling through PI3K suggest that Wit may act as a RTK rather than a Ser/Thr kinase receptor. C) Postsynaptic FGFR-like receptors. The heterozygous mutant conditions for muscle receptors Htl or Btl increase synapse number. This is consistent with a repressive role in the levels of Gbb and, thus, in the increased pro-synaptogenesis signaling (see also S2 Fig). Genotypes: Control = D42-Gal4/+. Cic↑ = D42-Gal4/UAS-cic. Htl↓ = htlAB42/+; D42-Gal4/+. Btl↓ = btldev1/+; D42-Gal4/+. Bar in A = 5 μm. Number of independent larvae analyzed is shown in parenthesis. A single NMJ from abdominal segment A3 was analyzed per larvae. (TIF) [file pone.0184238.s001.tif]

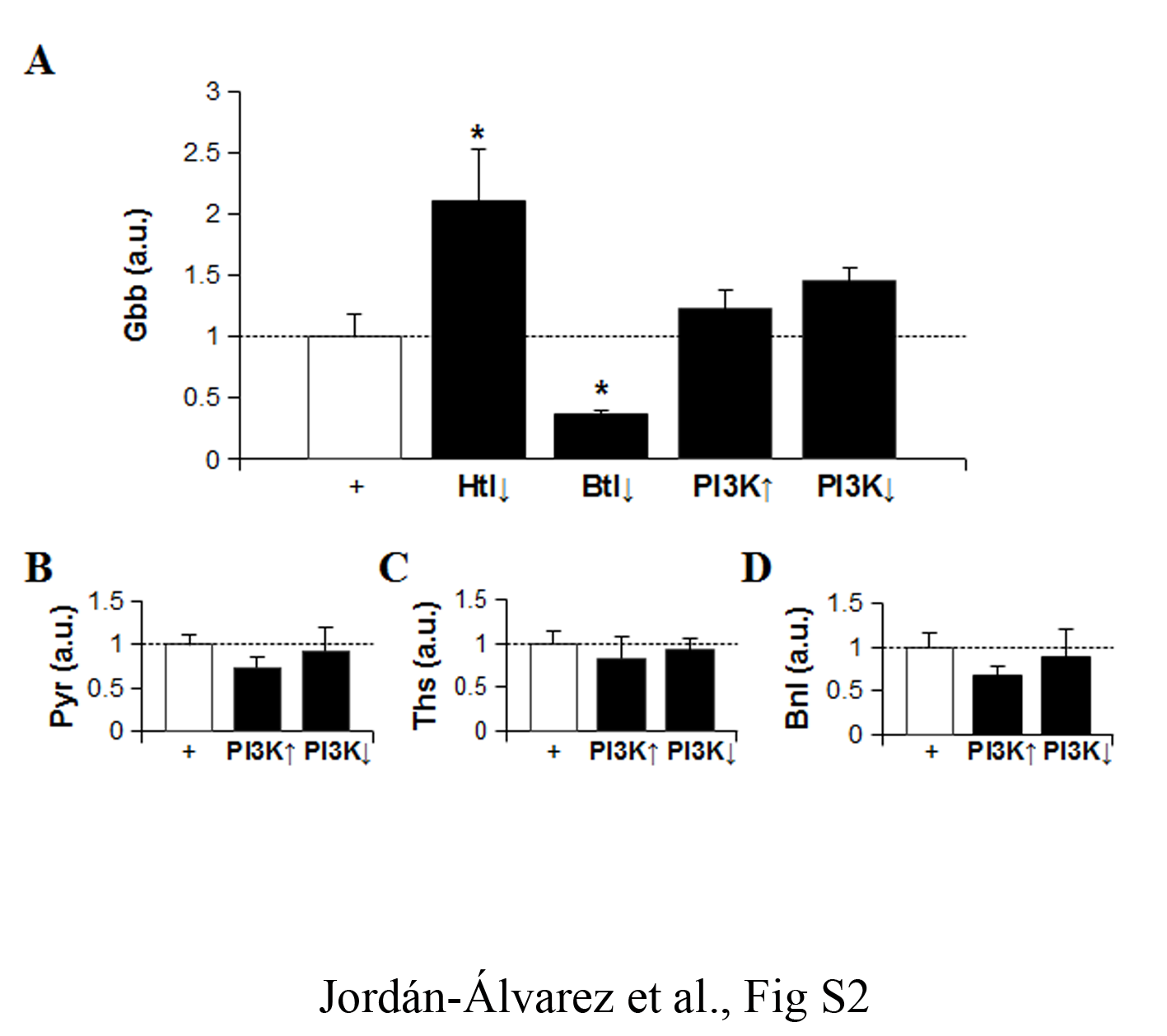

Supplement: S2 Fig — A) Based on q-PCR assays, Gbb expression is increased as a result of the haploinsufficiency of Htl receptor. By contrast, the haploinsufficiency of Btl receptor leads to the opposite effect illustrating their antagonistic regulation on Gbb expression. The up- or down-regulation of PI3K in neurons has no effect on Gbb transcription. This result indicates that PI3K does not alter gbb transcription in the neuron nor triggers a signaling that could have modified that transcription in the muscle (see main text). B-D) The neural manipulation of PI3K has no effect on the Htl ligands, Pyramus (Pyr) (B) and Thisbe (Ths) (C), or on the Btl ligand Branchless (Bnl) (D). Genotypes: + = elav-Gal4/+. Htl↓ = htlAB42/+. Btl↑ = btldev1/+. PI3K↑ = UAS-PI3K/+; elav-Gal4/+. PI3K↓ = UAS-PI3KDN/+; elav-Gal4/+. (TIF) [file pone.0184238.s002.tif]

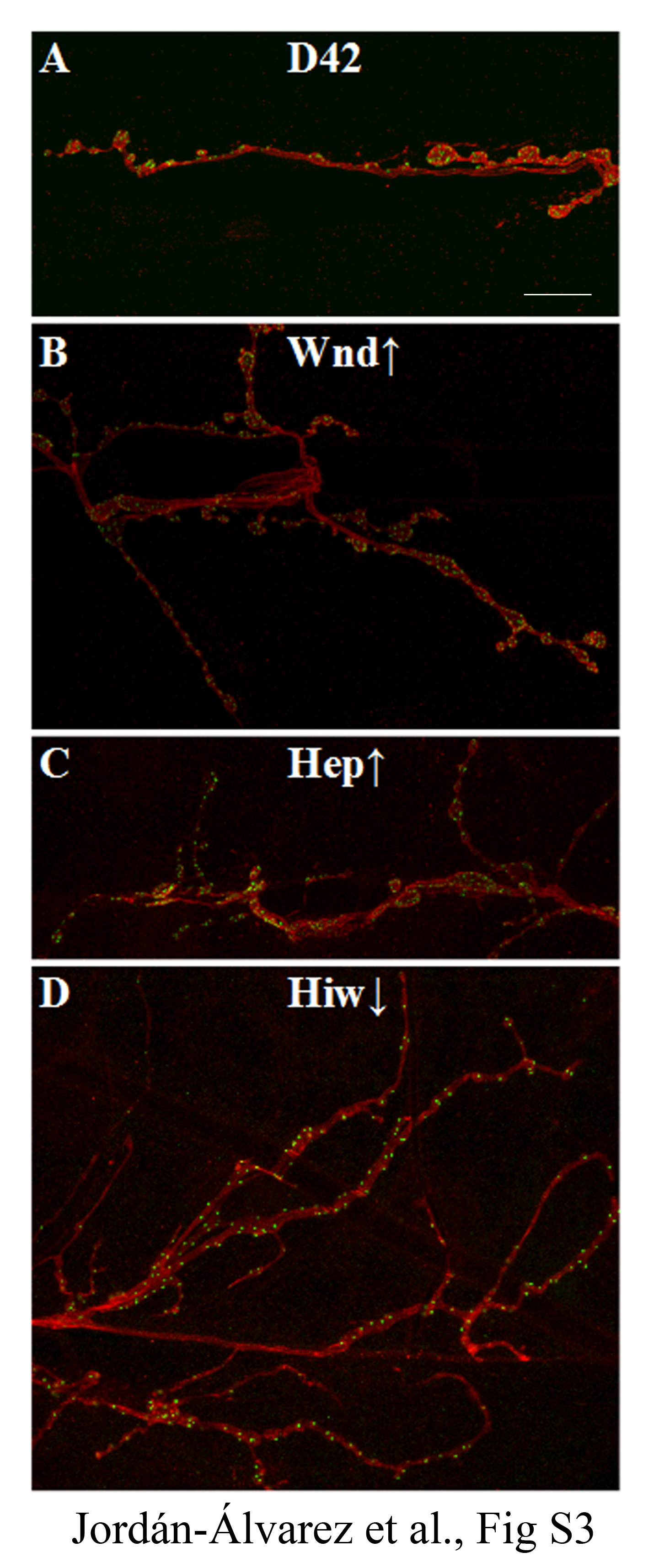

Supplement: S3 Fig — A-D) Representative images of NMJs. The number of active zones and the size of the motor neuron deviate in opposite directions in Hiw↓. Motor neuron branches are more extensive than control but have fewer synapses. Also, note the frequent location of synapses, nc82 puncta, outside of boutons, particularly in Hiw↓. See also S3 Table. Genotypes: A = D42-Gal4/+. B = UAS-Wnd/+; D42-Gal4/+. C = UAS-Hep/+; D42-Gal4/+. D = males hiwND8; +/+; D42-Gal4/+. Bar in A = 20 μm. (TIF) [file pone.0184238.s003.tif]
