# Supplementary material for: The equilibrium between antagonistic signaling pathways determines the number of synapses in Drosophila
Source: PLoS One. 2017 Sep 11;12(9):e0184238. doi: 10.1371/journal.pone.0184238 (PMC5593197; doi:10.1371/journal.pone.0184238)
Supplement: S1 Table — (DOCX) [file pone.0184238.s004.docx]

**Table S1**

**Receptors without effect in synaptogenesis**

| **NAME** | **REFERENCE** |
| --- | --- |
| Epidermal Growth Factor Receptor (EGFR) | Martín - Peña et al., 2006 |
| Insulin-like Receptor (InR) | Martín - Peña et al., 2006 |
| Anaplastic Lymphoma Kinase (Alk) | Rohrbough and Broadie, 2010 |
| Torso (tor) | This article |
| Off - track (Otk) | This article |
| Neurospecific Receptor Kinase (Nrk) | This article |
| Thick veins (Tk) | This article |
| Saxophone (Sax) | This article |
