# Supplementary material for: The equilibrium between antagonistic signaling pathways determines the number of synapses in Drosophila
Source: PLoS One. 2017 Sep 11;12(9):e0184238. doi: 10.1371/journal.pone.0184238 (PMC5593197; doi:10.1371/journal.pone.0184238)
Supplement: S3 Table — (DOC) [file pone.0184238.s006.doc]

| **Table S3. Number of boutons versus number of synapses** | | | |
| --- | --- | --- | --- |
| **Genotype** | **N** | **Number of boutons per NMJ (Difference)** | **Number of synapses (Difference)** |
| ***D42-Gal4/+*** | 8 | 83±5 |  |
| ***UAS-PI3K/+; D42-Gal4/+*** | 8 | 108±6 ( Δ1.30*) | Δ 1.72* |
| ***Hiw^ND8^;+/+; D42-Gal4/+*** | 8 | 93±8 (Δ1.12) | Δ 0.61 |

* = p<0.05 or higher
